# Risk factors for severe post-COVID condition in children, adolescents, and young adults

**DOI:** 10.1007/s00431-026-06995-3

**Published:** 2026-05-04

**Authors:** Quirin Donath, Matthias Haegele, Daniela Schindler, Tiziana Welzhofer, Catharina Christa, Annika Grabbe, Ariane Leone, Clara Ilhan, Carola Weidmann, Maria Eberhartinger, Sara Bechtold, Nicola Bursch, Hedwig Wolf, Hannah Hieber, Laura-Carlotta Peo, Lara A. Bucka, Silvia Stojanov, Cordula Warlitz, Martin Alberer, Katrin Gerrer, Anna Hausruckinger, Kirstin Mittelstrass, Clemens-Martin Wendtner, Manuela A. Hoechstetter, Armin Grübl, Nicole Toepfner, Rafael Pricoco, Carmen Scheibenbogen, Lorenz L. Mihatsch, Uta Behrends

**Affiliations:** 1 TUM School of Medicine and Health, Munich Chronic Fatigue Center for Young People (MCFC), Pediatrics, Children’s Hospital, Technical University of Munich, Munich, Germany; 2 German Center for Infection Research (DZIF), Munich, Germany; 3 TUM School of Medicine and Health, Munich Chronic Fatigue Center for Young People (MCFC), Child and Adolescent Psychosomatics, Children’s Hospital, Munich, Germany; 4 Division of Pediatrics Psychosomatic Medicine, Department of Pediatrics and Adolescent Medicine, KJF Klinikum Josefinum, Augsburg, Germany; 5 Medical Clinic III, Ludwig-Maximilians University (LMU), Munich, Germany; 6 Department of Pediatrics, Faculty of Medicine, University Hospital Carl Gustav Carus, Technische Universität Dresden, Dresden, Germany; 7 Institute of Medical Immunology, Charité-Universitaetsmedizin Berlin, Corporate Member of Freie Universität Berlin and Humboldt Universität Zu Berlin and Berlin Institute of Health (BIH), Berlin, Germany

**Keywords:** Post-COVID syndrome (PCS), Post-COVID-19 condition (PCC), Long COVID, Post-acute sequelae of COVID-19 (PASC), Myalgic encephalomyelitis/chronic fatigue syndrome (ME/CFS), Post-exertional malaise (PEM)

## Abstract

Post-COVID condition (PCC) in children and young people (CYP, PCCcyp) remains a significant health burden. Early identification of patients at risk for severe disease, including myalgic encephalomyelitis/chronic fatigue syndrome (ME/CFS), is crucial for timely and adequate care. This monocentric, observational registry study, performed at a tertiary pediatric hospital in Germany, included CYP aged 7–25 years with PCCcyp at diagnosis. Standardized clinical assessment tools and patient-reported outcome measures were applied, including the novel Munich Long COVID Symptom Questionnaire (MLCSQ). Severe PCC was defined by chronic symptom clusters, Fatigue Severity Scale (FSS), Total Composite Autonomic Symptom Score-31 (COMPASS-31), SF-36 composite scores, Bell Score, and confirmed ME/CFS diagnosis. Among 120 participants, severe PCCcyp was associated with a higher number of acute symptoms (OR_adj_ 1.22, *P* < 0.001), acute orthostatic intolerance (OR_adj_ 9.87, *P* = 0.002), acute trouble concentrating (OR_adj_ 11.8, *P* = 0.005), and female sex (OR 3.31, *P* = 0.031). Categorizing acute symptoms at a threshold of ≥ 12 yielded optimal model performance (AUC 0.857; sensitivity 65.6%; specificity 90.2%). ME/CFS was diagnosed in 24% of participants, all within the severe PCCcyp cluster, and was characterized by greater acute symptom complexity, more fatigue, more autonomic symptoms, and poorer function.

*Conclusions*: The number and pattern of acute symptoms during SARS-CoV-2 infection may serve as early, specific predictors of severe PCCcyp. Patients with ≥ 12 acute symptoms should be closely monitored to enable early diagnosis of severe PCCcyp and ME/CFS. A distinct cluster of severely affected patients, frequently with ME/CFS, was identified.

*Trial registration*: ClinicalTrials.gov: NCT05638724; Ethics approval (511/21, 2025–465-S-SB).

**Supplementary Information:**

The online version contains supplementary material available at 10.1007/s00431-026-06995-3.

**Table:** 

**What is Known:** • *Post-COVID condition in children and young people (PCCcyp) is increasingly reported, yet clinicians lack reliable markers to identify patients at risk of severe disease early on*.		
**What is New:** • *Severe PCCcyp in pediatric patients can be predicted during the acute phase of the SARS-CoV-2 infection*. • *A high acute symptom load (≥ 12 symptoms), female sex, acute orthostatic intolerance, and acute trouble concentrating predict severe PCCcyp*. • *24% of patients fulfilled ME/CFS criteria—all within the severe cluster—highlighting a distinct high-risk subgroup that necessitates early stratification and care*.		

## Introduction

Post-acute sequelae of coronavirus disease 2019 (COVID-19) (PASC), often termed Long COVID, represent major clinical and economic challenges worldwide [1–7]. PASC comprises ongoing, relapsing, or new symptoms occurring ≥ 4 weeks after infection with SARS-CoV-2 [8]. A chronic form has been defined by the WHO as post-COVID condition (PCC) [9]. In children and adolescents, PCC is defined as symptoms lasting ≥ 2 months that began within 3 months of acute COVID-19 in individuals with confirmed or probable SARS-CoV-2 infection [4].

PCC can cause symptoms across all organ systems and often significantly impacts functioning, participation, and health-related quality of life (HRQoL) [10, 11]. Frequent symptoms include fatigue, exertional intolerance, headache, respiratory symptoms, cognitive dysfunction, including brain fog, sleep problems, loss of smell and taste, anxiety, and autonomic dysfunction [12–18]. The latter can manifest as orthostatic intolerance (OI) with or without postural orthostatic tachycardia syndrome (PoTS) [19]. Some patients develop post-exertional malaise (PEM), characterized by symptom worsening after previously tolerated activities. Long-lasting PEM is a cardinal symptom of myalgic encephalomyelitis/chronic fatigue syndrome (ME/CFS), a chronic multisystemic disease defined by clinical criteria and mostly triggered by viral infections, with a presumed multifactorial etiology, including immunodysregulation, genetics, and possibly environmental factors [8, 19–22]. Recent studies estimate ME/CFS prevalences of 4.5 to 11.6% among adult PCC patients, depending on study design [23, 24].

Data on PCC in CYP (PCCcyp) remain scarce, and age-specific factors are insufficiently understood [8]. Reported PASC prevalence in minors ranges from 0.8 to 60% [3, 11, 15, 25], with lower estimates in studies including SARS-CoV-2-negative controls [11, 26–32]. While most CYP recover within months, a subset develops long-term disease [5, 13, 15, 30, 33–35].

In adults, established PCC risk factors include infection with pre-Omicron variants [5, 6], older age, female sex, pre-existing chronic conditions (such as atopic, psychiatric, and autoimmune diseases), lack of COVID-19 vaccination [36], more severe acute illness, and greater symptom complexity [37]. Preliminary pediatric data suggest similar patterns, but evidence remains limited and heterogeneous [16, 25, 38–43].

In the IMMUC study of EBV-associated infectious mononucleosis in CYP, susceptibility to infection, higher acute symptom burden, and early gastrointestinal (GI) involvement predicted prolonged recovery and chronic fatigue [44]. Pre-existing GI symptoms, prior to infection, have also been linked to severe ME/CFS [47].

Here, we characterize acute and chronic clinical phenotypes after SARS-CoV-2 infection in 120 PCCcyp patients, including patients with post-COVID ME/CFS, and identify clinical risk factors for severe PCCcyp that may support earlier recognition and care.

## Materials and methods

### Study cohort and case definition

This monocentric observational registry study included 120 CYP aged 7 to 25 years and diagnosed with PCC according to WHO definitions [9] between January 2021 and May 2024 at the Munich Chronic Fatigue Center for Young People (MCFC). This age range is supported by ME/CFS epidemiology and the clinical continuity of severe PCC across adolescence and early adulthood [21].

Confirmed SARS-CoV-2 infection was defined by a positive PCR or rapid antigen test with typical COVID-19 symptoms. Probable infection was defined by COVID-19 symptoms after contact with a PCR-positive person and a positive SARS-CoV-2 serology not explained by vaccination.

All patients underwent a symptom-oriented diagnostic work-up including a comprehensive, structured, multiprofessional medical history, pulmonary function testing, electrocardiography, echocardiography, orthostatic assessment using the NASA Lean Test, and laboratory analyses, to exclude alternative somatic and psychiatric conditions [46, 49].

Confirmed ME/CFS was defined by fulfilling the criteria of the Institute of Medicine (IOM) [48] and/or the Canadian Consensus Criteria (CCC) [50], assessed using the Munich Berlin Symptom Questionnaire (MBSQ) [19, 20] in a structured medical interview after symptom-oriented differential diagnosis. In pediatric patients, the pediatric case definition by Jason et al. (PCD-J) [51] and/or the clinical diagnostic worksheet by Rowe et al. (CDW-R) [21] were additionally used.

### Assessed data

Patients completed questionnaires retrospectively covering general medical history, acute SARS-CoV-2 symptoms, current symptoms, medical treatment, participation in education/work, and HRQoL. To assess PCC symptoms, ME/CFS criteria, fatigue severity, PEM, autonomic symptoms, daily activity, and HRQoL, we used the Munich Long COVID Symptom Questionnaire (MLCSQ), MBSQ [19, 20], Fatigue Severity Scale (FSS) [52], DePaul Symptom Questionnaire for PEM (DSQ-PEM) [53], Composite Autonomic Symptom Score 31 (COMPASS 31) [54], Bell Score [55], and the Short Form-36 Health Survey (SF-36; PCS and MCS) [56, 57].

The MLCSQ was developed for structured age-independent assessment of PCC symptoms in 13 domains, encompassing 83 items (Supplementary Material M1). Items were adapted from the WHO Global COVID-19 Clinical Platform Case Report Form (2021) [58], the DGPI Long COVID-19 Survey (2021) [59], and COMPASS 31 [54]. Frequency and severity were rated on Likert scales. Only symptoms rated as at least intermittent or persistent were counted as PCC symptoms.

### Statistical analysis

#### Descriptive analysis

Continuous variables are reported as median (IQR) and compared using Kruskal–Wallis or Wilcoxon rank-sum tests. Categorical variables are reported as *n*/*N* (%) and compared using chi-squared or Fisher’s exact tests, as appropriate. A *P*-value < 0.05 was considered statistically significant.

#### Risk factor analysis using distinct clinical outcomes

Multivariable linear regression models (SF36-PCS, SF36-MCS, number of PCC symptoms) and logistic regression models (PEM, ME/CFS, PoTS), adjusted for sex and age, were fitted. Candidate predictors were vaccination status [36], hospitalization during COVID-19 [60], number of acute symptoms [47], acute GI symptoms [45], acute OI symptoms [61], acute fatigue [62], and trouble concentrating [15].

#### Identification and risk factor analysis for severe PCC cluster

A data-driven clustering approach was applied to identify patient subgroups based on PCC severity. The following outcome variables were used for the cluster definition: Bell Score, FSS, COMPASS 31 total score, SF36-PCS, SF36-MCS, number of PCC symptoms, ME/CFS diagnosis (ICD-10 G93.3). *K*-means clustering (*k* = 2) was applied to standardized data from patients without missing values in these variables (70/120, 58%). The resulting clusters represented moderate and severe PCC. Cluster membership served as the primary outcome in subsequent analyses. As sensitivity analyses, we repeated the clustering excluding ME/CFS diagnosis and quantified concordance (percent agreement, adjusted Rand index (ARI)) and cluster stability by bootstrap resampling (*B* = 500; Jaccard similarity).

Using LASSO-penalized logistic regression, we assessed associations between acute-phase COVID-19 features and later cluster membership using the same candidate predictors as above. The model was fitted with tenfold cross-validation to select the penalty parameter (λ). To account for class imbalance, inverse class weights were applied. Resulting coefficients of the cross-validated model can be used to calculate a predicted probability $P= \left(\frac{1}{1+{e}^{-z}}\right),$ for being attributed to the severe PCC cluster as, where $z={predictor}_{1}\times {coefficient}_{1}+\dots +{predictor}_{q}\times {coefficient}_{q}$ and $q$ the number of selected predictors by the LASSO operator.

Model performance was assessed by discrimination and calibration (AUC, calibration plot, calibration intercept/slope, and Brier score). Internal validation used bootstrap resampling (B = 500), repeating the full modeling procedure, including cross-validation for λ selection. Optimism-corrected performance measures are shown in Supplementary Figure S1. The optimal classification threshold was determined using the Youden index. Sensitivity and specificity were reported for all acute symptom count cut-offs.

To explore recall bias from retrospective reporting, we tested associations between time-to-diagnosis and retrospectively reported acute-phase predictors in age-adjusted and sex-adjusted models, and repeated the primary severity-cluster models with additional adjustment for time-to-diagnosis.

All analyses used complete cases to avoid additional imputation assumptions for multi-item PROM summary scores. Missingness and effective sample sizes are reported. Analyses were performed using R 4.2.1.

## Results

### Patient characteristics

Among 120 CYP with PCC, 16/120 (13%) were children (7–11 years), 71/120 (59%) adolescents (12–17 years), and 33/120 (28%) young adults (18–25 years). The proportion of females increased significantly with age (Table 1).

**Table 1:** Patients’ basic characteristics, vaccination status, and symptoms during the acute phase of PCC-related SARS-CoV-2 infection

	Overall^a^ *N* = 120	Childrenᵃ Age 7 to 11 years *N* = 16	Adolescentsᵃ Age 12 to 17 years *N* = 71	Young adultsᵃ Age 18 to 25 years *N* = 33	*P*-value^b^
Age^a^, years	15.0 (13.0–18.0)	11.0 (9.8–11.0)	15.0 (13.0–16.0)	21.0 (19.0–23.0)	
Sex, female	71/120 (59%)	2/16 (13%)	44/71 (62%)	25/33 (76%)	< 0.001
Body height^a^, cm	167.0 (160.8–173.8)	146.0 (141.8–152.3)	167.0 (161.8–173.3)	170.1 (166.5–177.0)	
Body weight^a^, kg	58.5 (48.4–68.6)	35.0 (31.7–45.8)	60.7 (50.9–68.6)	62.5 (56.6–75.0)	
Body mass index^a^, kg/m^2^	20.7 (18.4–22.9)	17.1 (15.9–19.0)	21.3 (18.8–22.9)	21.1 (19.5–24.7)	< 0.001
Allergies	51/116 (44%)	6/15 (40%)	29/69 (42%)	16/32 (50%)	0.714
Allergies since PCC onset	4/39 (10%)	1/2 (50%)	2/22 (9.1%)	1/15 (6.7%)	0.214
Susceptibility to infection^c^	26/110 (24%)	2/14 (14%)	15/65 (23%)	9/31 (29%)	0.614
Standard vaccination according to STIKO	93/95 (98%)	12/13 (92%)	59/60 (98%)	22/22 (100%)	0.308
COVID-19 vaccinations before symptom onset					0.007
None	82/116 (71%)	14/16 (88%)	40/69 (58%)	28/31 (90%)	
1	0/116 (0%)	0/16 (0%)	0/69 (0%)	0/31 (0%)	
2	26/116 (22%)	2/16 (13%)	21/69 (30%)	3/31 (10%)	
3	8/116 (7%)	0/16 (0%)	8/69 (12%)	0/31 (0%)	
PCC-triggering SARS-CoV-2 infection					0.430
1st	112/120 (93%)	14/16 (88%)	66/71 (93%)	32/33 (97%)	
2nd	8/120 (7%)	2/16 (13%)	5/71 (7%)	1/33 (3%)	
Documentation of SARS-CoV-2 infection					0.166
PCR	106/120 (88%)	14/16 (88%)	61/71 (86%)	31/33 (94%)	
Rapid antigen test	8/120 (7%)	2/16 (13%)	6/71 (9%)	0/33 (0%)	
Serology	6/120 (5%)	0/16 (0%)	4/71 (6%)	2/33 (6%)	
Time from infection to PCC symptom onset, days	0.5 (−0.3–30.8)	0.0 (−0.3–29.5)	12.0 (0.0–38.5)	0.0 (−3.0–8.0)	0.005
Time from infection to diagnosis, months	8.2 (5.4–12.4)	6.5 (5.6–8.8)	7.5 (5.0–12.1)	10.8 (7.9–14.5)	0.007
Acute symptom treatment					0.806
No medical visit	85/117 (73%)	12/16 (75%)	48/68 (71%)	25/33 (76%)	
Outpatient	23/117 (20%)	4/16 (25%)	13/68 (19%)	6/33 (18%)	
Hospitalized	9/117 (8%)	0/16 (0%)	7/68 (10%)	2/33 (6%)	
Number of acute symptoms reported	16.0 (11.0–21.0)	17.0 (13.0–18.5)	14.5 (11.0–20.0)	16.0 (12.0–23.0)	0.580
Acute symptoms					
Fatigue	104/116 (90%)	15/15 (100%)	60/68 (88%)	29/33 (88%)	0.503
Headaches	103/116 (89%)	13/15 (87%)	60/68 (88%)	30/33 (91%)	0.918
Sore throat	91/116 (78%)	14/15 (93%)	55/68 (81%)	22/33 (67%)	0.107
Persistent dry cough	87/116 (75%)	11/15 (73%)	54/68 (79%)	22/33 (67%)	0.375
Trouble in concentrating	85/116 (73%)	13/15 (87%)	47/68 (69%)	25/33 (76%)	0.415
Shortness of breath	84/116 (72%)	11/15 (73%)	50/68 (74%)	23/33 (70%)	0.955
Cold	83/116 (72%)	9/15 (60%)	53/68 (78%)	21/33 (64%)	0.184
Dizziness	75/116 (65%)	9/15 (60%)	43/68 (63%)	23/33 (70%)	0.752
Muscle pain	70/116 (60%)	8/15 (53%)	43/68 (63%)	19/33 (58%)	0.722
Fever	65/116 (56%)	11/15 (73%)	38/68 (56%)	16/33 (48%)	0.274
Loss of appetite	61/116 (53%)	13/15 (87%)	33/68 (49%)	15/33 (45%)	0.017
Chest tightness	59/116 (51%)	9/15 (60%)	30/68 (44%)	20/33 (61%)	0.224
Joint pain	59/116 (51%)	7/15 (47%)	34/68 (50%)	18/33 (55%)	0.859
Nausea	53/116 (46%)	8/15 (53%)	34/68 (50%)	11/33 (33%)	0.235
Stomach pain	51/116 (44%)	10/15 (67%)	28/68 (41%)	13/33 (39%)	0.163
Pain on breathing	47/116 (41%)	6/15 (40%)	24/68 (35%)	17/33 (52%)	0.297
Palpitations	45/116 (39%)	6/15 (40%)	23/68 (34%)	16/33 (48%)	0.364
Tachycardia	45/116 (39%)	4/15 (27%)	22/68 (32%)	19/33 (58%)	0.030
Swollen lymph nodes	44/116 (38%)	8/15 (53%)	27/68 (40%)	9/33 (27%)	0.202
Loss of smell	44/116 (38%)	0/15 (0%)	25/68 (37%)	19/33 (58%)	< 0.001
Apathy	43/116 (37%)	5/15 (33%)	22/68 (32%)	16/33 (48%)	0.275
Light-headedness	42/116 (36%)	4/15 (27%)	24/68 (35%)	14/33 (42%)	0.558
Weight loss	38/116 (33%)	5/15 (33%)	21/68 (31%)	12/33 (36%)	0.883
Loss of taste	38/116 (33%)	0/15 (0%)	23/68 (34%)	15/33 (45%)	0.003
Altered taste	32/116 (28%)	4/15 (27%)	19/68 (28%)	9/33 (27%)	> 0.999
Altered smell	29/116 (25%)	3/15 (20%)	17/68 (25%)	9/33 (27%)	0.909
Breath-independent chest pain	28/116 (24%)	6/15 (40%)	12/68 (18%)	10/33 (30%)	0.100
Numbness or tingling	26/116 (22%)	5/15 (33%)	14/68 (21%)	7/33 (21%)	0.561
Diarrhea	25/116 (22%)	9/15 (60%)	9/68 (13%)	7/33 (21%)	< 0.001
Fainting/blackouts	14/116 (12%)	1/15 (7%)	11/68 (16%)	2/33 (6%)	0.393
Vomiting	13/116 (11%)	2/15 (13%)	7/68 (10%)	4/33 (12%)	0.841
Conjunctivitis	11/116 (10%)	0/15 (0%)	7/68 (10%)	4/33 (12%)	0.483
Skin rash	9/116 (8%)	2/15 (13%)	3/68 (4%)	4/33 (12%)	0.226
Pain in the limbs	7/116 (6%)	0/15 (0%)	3/68 (4%)	4/33 (12%)	0.252
COVID-19 toes (purple, pink, bluish)	6/116 (5%)	0/15 (0%)	5/68 (7%)	1/33 (3%)	0.585

*PCR* polymerase chain reaction, *SARS-CoV-2* severe acute respiratory syndrome coronavirus 2, *STIKO* Standing Committee on Vaccination at the Robert Koch Institute (Germany)

^a^Data are expressed as *n*/*N* (%), median (IQR), or mean (SD), as appropriate. Percentages may not total 100% because of rounding

^b^*P*-values were calculated using the *χ*^2^ test, Fisher exact test, or Kruskal–Wallis test, as appropriate

^c^Susceptibility to infection was defined as ≥ 1 of the following: recurrent infections at the same site, > 2 antibiotic courses/year, hospitalization due to infection, ≥ 4 episodes of herpes labialis/year, and at least one episode of genital herpes or herpes zoster

### Characteristics of the acute phase of the SARS-CoV-2 infection

Documented or probable SARS-CoV-2 infections occurred between February 2020 and January 2023, covering German pandemic waves dominated by Alpha (43/114 (38%)), Delta (25/114 (22%)), and Omicron variants (46/114 (40%)) [63].

In 112/120 (93%) patients, the first SARS-CoV-2 infection triggered PCC, while 8/120 (7%) reported the second infection as the trigger. The triggering infection was documented by PCR in 106/120 (88%), rapid antigen testing in 8/120 (7%), or serology combined with contact history in 6/120 (5%). Before the index infection, 82/116 (71%) were not COVID-19. A triggering breakthrough infection was reported in 34/116 (29%) patients (Table 1).

Patients reported a median of 16 acute symptoms (IQR 11–21), without age-group differences (*P* = 0.579). The most frequent symptoms were fatigue, headaches, sore throat, persistent dry cough, and trouble concentrating (Table 1). Loss of appetite (*P* = 0.017) and diarrhea (*P* < 0.001) were more frequent in children, whereas heart racing (*P* = 0.030), loss of smell (*P* < 0.001), and loss of taste (*P* = 0.003) were more frequent in adolescents and young adults. Overall, 9/117 (8%) patients were hospitalized (Table 1).

### Characteristics of post-COVID-condition

Median time from acute symptom onset to diagnosis was 8.2 months (IQR 5.4–12.4) and was longer in young adults than in children and adolescents (*P* = 0.007). Patients presented with various PCC-associated diagnoses (Suppl. Table S1**)**, most commonly fatigue (ICD-10-GM R53; 81/120, 68%), brain fog (ICD-10-GM F06.7; 64/120, 53%), and headaches (ICD-10-GM R51; 49/120, 41%). Brain fog was more frequent in young adults (*P* = 0.034).

The median number of PCC symptoms at diagnosis was 29 (IQR 21–37); the most frequent were fatigue, exercise intolerance, reduced endurance, trouble concentrating, and headaches (Table 2). Median FSS was 6.4 (IQR 5.8–6.6) (Table 3). PEM was present in 112/119 (94%) patients, with durations of 14–23 h and ≥ 24 h in 19/114 (17%) and 23/114 (20%), respectively (Table 3). Median COMPASS 31 OI score was 20.0 (IQR 12.0–28.0), and median total score was 27.8 (IQR 17.8–38.8), indicating severe autonomic distress (Table 3).

**Table 2:** Number of chronic postviral symptoms assessed by the Munich Long COVID Symptom Questionnaire (MLCSQ)

	Overall^a^ *N* = 120	Children^a^ Age 7 to 11 years *N* = 16	Adolescents^a^ Age 12 to 17 years *N* = 71	Young adults^a^ Age 18 to 25 years *N* = 33	*P*-value^b^
Number of 83 possible PCC symptoms reported	29.0 (21.0–37.0)	29.5 (18.0–35.25)	29.0 (20.0–36.0)	31.0 (24.5–37.75)	0.448
Fatigue/exercise intolerance					
Fatigue	105/109 (96%)	14/16 (88%)	63/65 (97%)	28/28 (100%)	0.097
Worsening of symptoms following mild mental and/or physical activity (exercise intolerance)	104/109 (95%)	14/16 (88%)	62/65 (95%)	28/28 (100%)	0.162
Reduced endurance	103/109 (94%)	14/16 (88%)	61/65 (94%)	28/28 (100%)	0.203
Neurological					
Trouble in concentrating	97/109 (89%)	15/16 (94%)	55/65 (85%)	27/28 (96%)	0.200
Headaches	96/109 (88%)	14/16 (88%)	56/65 (86%)	26/28 (93%)	0.656
Dizziness	84/109 (77%)	10/16 (62%)	54/65 (83%)	20/28 (71%)	0.153
Forgetfulness	81/109 (74%)	11/16 (69%)	47/65 (72%)	23/28 (82%)	0.523
Light-headedness	54/109 (50%)	3/16 (19%)	33/65 (51%)	18/28 (64%)	0.014
Problems with balance	50/109 (46%)	6/16 (38%)	33/65 (51%)	11/28 (39%)	0.456
Numbness or tingling	34/109 (31%)	7/16 (44%)	17/65 (26%)	10/28 (36%)	0.331
Slowness of movement	28/109 (26%)	5/16 (31%)	16/65 (25%)	7/28 (25%)	0.858
Problems with gait/falls	21/109 (19%)	4/16 (25%)	16/65 (25%)	1/28 (4%)	0.051
Seizures	8/109 (7%)	1/16 (6%)	6/65 (9%)	1/28 (4%)	0.621
Can not feel one side of body or face	7/109 (6%)	0/16 (0%)	6/65 (9%)	1/28 (4%)	0.312
Can not move one side of body or face	1/109 (1%)	0/16 (0%)	0/65 (0%)	1/28 (4%)	0.232
Pulmonary					
Shortness of breath with activity	88/109 (81%)	12/16 (75%)	54/65 (83%)	22/28 (79%)	0.722
Persistent dry cough	48/109 (44%)	7/16 (44%)	30/65 (46%)	11/28 (39%)	0.829
Shortness of breath at rest	44/109 (40%)	4/16 (25%)	30/65 (46%)	10/28 (36%)	0.256
Pain on breathing	43/109 (39%)	3/16 (19%)	24/65 (37%)	16/28 (57%)	0.035
Congestion	36/109 (33%)	2/16 (12%)	24/65 (37%)	10/28 (36%)	0.167
Cardiovascular					
Orthostatic intolerance	83/109 (76%)	6/16 (38%)	53/65 (82%)	24/28 (86%)	< 0.001
Heart pounding	55/109 (50%)	6/16 (38%)	31/65 (48%)	18/28 (64%)	0.181
Chest tightness	54/109 (50%)	4/16 (25%)	30/65 (46%)	20/28 (71%)	0.009
Heart racing	51/109 (47%)	5/16 (31%)	30/65 (46%)	16/28 (57%)	0.251
Breath-independent chest pain	25/109 (23%)	3/16 (19%)	13/65 (20%)	9/28 (32%)	0.403
Fainting and/or Blackouts	16/109 (15%)	1/16 (6%)	15/65 (23%)	0/28 (0%)	0.009
Sleep					
Sleeping more	81/109 (74%)	10/16 (62%)	47/65 (72%)	24/28 (86%)	0.201
Other sleep disturbances	50/109 (46%)	7/16 (44%)	30/65 (46%)	13/28 (46%)	0.983
Sleeping less	37/109 (34%)	3/16 (19%)	22/65 (34%)	12/28 (43%)	0.267
Mental health					
Listlessness/lack of motivation	80/109 (73%)	11/16 (69%)	50/65 (77%)	19/28 (68%)	0.597
Loss of interest/pleasure	61/109 (56%)	7/16 (44%)	37/65 (57%)	17/28 (61%)	0.535
Depressed mood	59/109 (54%)	5/16 (31%)	37/65 (57%)	17/28 (61%)	0.131
Anxiety	44/109 (40%)	7/16 (44%)	25/65 (38%)	12/28 (43%)	0.884
Behavioral change	35/109 (32%)	5/16 (31%)	20/65 (31%)	10/28 (36%)	0.893
Tic	11/109 (10%)	2/16 (12%)	8/65 (12%)	1/28 (4%)	0.414
Hallucinations	6/109 (6%)	1/16 (6%)	5/65 (8%)	0/28 (0%)	0.325
Musculoskeletal					
Muscle pain	69/109 (63%)	12/16 (75%)	38/65 (58%)	19/28 (68%)	0.397
Muscle weakness	63/109 (58%)	9/16 (56%)	35/65 (54%)	19/28 (68%)	0.451
Back pain	59/109 (54%)	9/16 (56%)	33/65 (51%)	17/28 (61%)	0.666
Joint pain	52/109 (48%)	9/16 (56%)	29/65 (45%)	14/28 (50%)	0.678
Muscle twitching	36/109 (33%)	4/16 (25%)	21/65 (32%)	11/28 (39%)	0.614
Muscle tremor	34/109 (31%)	3/16 (19%)	23/65 (35%)	8/28 (29%)	0.411
Muscle stiffness	17/109 (16%)	3/16 (19%)	7/65 (11%)	7/28 (25%)	0.207
Joint swelling	5/109 (5%)	2/16 (12%)	2/65 (3%)	1/28 (4%)	0.260
Redness and/or overheating of joints	4/109 (4%)	0/16 (0%)	3/65 (5%)	1/28 (4%)	0.679
Gastrointestinal					
Stomach pain	65/109 (60%)	10/16 (62%)	37/65 (57%)	18/28 (64%)	0.777
Nausea	55/109 (50%)	10/16 (62%)	30/65 (46%)	15/28 (54%)	0.468
Loss of appetite	48/109 (44%)	11/16 (69%)	25/65 (38%)	12/28 (43%)	0.091
Diarrhea	39/109 (36%)	7/16 (44%)	20/65 (31%)	12/28 (43%)	0.414
Flatulence	38/109 (35%)	8/16 (50%)	19/65 (29%)	11/28 (39%)	0.251
Bloatedness	29/109 (27%)	4/16 (25%)	13/65 (20%)	12/28 (43%)	0.072
Constipation	25/109 (23%)	5/16 (31%)	10/65 (15%)	10/28 (36%)	0.070
Weight loss	16/109 (15%)	2/16 (12%)	9/65 (14%)	5/28 (18%)	0.851
Vomiting	6/109 (6%)	1/16 (6%)	4/65 (6%)	1/28 (4%)	0.873
Body temperature					
Increased sweating	63/109 (58%)	11/16 (69%)	39/65 (60%)	13/28 (46%)	0.301
Chills	34/109 (31%)	6/16 (38%)	18/65 (28%)	10/28 (36%)	0.627
Fever > 38.0 °C	18/109 (16%)	5/16 (31%)	8/65 (12%)	5/28 (18%)	0.184
Ears-nose-throat (ENT)					
Cold	46/109 (42%)	6/16 (38%)	29/65 (45%)	11/28 (39%)	0.820
Sore throat	40/109 (37%)	7/16 (44%)	24/65 (37%)	9/28 (32%)	0.743
Swollen lymph nodes	27/109 (25%)	6/16 (38%)	14/65 (22%)	7/28 (25%)	0.416
Dryness of the mouth	27/109 (25%)	2/16 (12%)	17/65 (26%)	8/28 (29%)	0.455
Altered taste	24/109 (22%)	5/16 (31%)	10/65 (15%)	9/28 (32%)	0.127
Problem swallowing	24/109 (22%)	5/16 (31%)	14/65 (22%)	5/28 (18%)	0.581
Ringing in ears	24/109 (22%)	4/16 (25%)	15/65 (23%)	5/28 (18%)	0.816
Altered smell	22/109 (20%)	3/16 (19%)	10/65 (15%)	9/28 (32%)	0.179
Ear pain	22/109 (20%)	3/16 (19%)	15/65 (23%)	4/28 (14%)	0.618
Sinus disorder	20/109 (18%)	1/16 (6%)	13/65 (20%)	6/28 (21%)	0.395
Voice disorder	18/109 (16%)	2/16 (12%)	12/65 (18%)	4/28 (14%)	0.792
Loss of smell	14/109 (13%)	0/16 (0%)	8/65 (12%)	6/28 (21%)	0.121
Hearing impairment	12/109 (11%)	3/16 (19%)	7/65 (11%)	2/28 (7%)	0.494
Loss of taste	9/109 (8%)	1/16 (6%)	4/65 (6%)	4/28 (14%)	0.405
Ophthalmic					
Light sensitivity	44/109 (40%)	7/16 (44%)	28/65 (43%)	9/28 (32%)	0.588
Dry eyes	29/109 (27%)	2/16 (12%)	17/65 (26%)	10/28 (36%)	0.243
Vision impairment	24/109 (22%)	5/16 (31%)	14/65 (22%)	5/28 (18%)	0.581
Conjunctivitis	24/109 (22%)	4/16 (25%)	13/65 (20%)	7/28 (25%)	0.826
Urogenital					
Painful menstruation	29/109 (27%)	0/16 (0%)	19/65 (29%)	10/28 (36%)	0.027
Problems passing urine	7/109 (6%)	1/16 (6%)	2/65 (3%)	4/28 (14%)	0.129
Changes in sexual function	1/109 (1%)	0/16 (0%)	0/65 (0%)	1/28 (4%)	0.232
Dermatological					
Hair loss	26/109 (24%)	0/16 (0%)	14/65 (22%)	12/28 (43%)	0.005
Skin rash	19/109 (17%)	3/16 (19%)	8/65 (12%)	8/28 (29%)	0.164
COVID-19 toes (purple, pink, bluish)	14/109 (13%)	0/16 (0%)	10/65 (15%)	4/28 (14%)	0.248
Other changes in skin color on hands and/or feet (red, white)	14/109 (13%)	2/16 (12%)	6/65 (9%)	6/28 (21%)	0.272
Swollen ankles^/^oedema	6/109 (6%)	0/16 (0%)	3/65 (5%)	3/28 (11%)	0.288

*MLCSQ* Munich Long COVID Symptom Questionnaire, *PCC* post-COVID condition

^**a**^Data are expressed as *n*/*N* (%) or median (IQR), as appropriate. Percentages may not total 100% because of rounding

^**b**^*P*-values were calculated using the *χ*^2^ test or Kruskal–Wallis test, as appropriate

**Table 3:** Characteristics of post-COVID condition assessed by questionnaires and defined by specific ICD-10 diagnoses

	Overall^a^ *N* = 120	Children^a^ Age 7 to 11 years *N* = 16	Adolescents^a^ Age 12 to 17 years *N* = 71	Young adults^a^ Age 18 to 25 years *N* = 33	*P*-value^b^
Participation					
Full participation	17/119 (14%)	1/16 (6%)	11/70 (16%)	5/33 (15%)	0.869
> 50% participation	33/119 (28%)	7/16 (44%)	18/70 (26%)	8/33 (24%)	
< 50% participation	40/119 (34%)	5/16 (31%)	24/70 (34%)	11/33 (33%)	
No participation	29/119 (24%)	3/16 (19%)	17/70 (24%)	9/33 (27%)	
Bell CFIDS disability scale (Bell Score)	40.0 (30.0–60.0)	50.0 (40.0–60.0)	40.0 (30.0–50.0)	50.0 (30.0–60.0)	0.111
Fatigue Severity Scale (FSS)	6.4 (5.8–6.6)	6.4 (5.5–6.6)	6.4 (6.0–6.6)	6.3 (5.5–6.6)	0.656
DePaul University Symptom Questionnaire for Post-exertional Malaise (DSQ-PEM)					
PEM screening positive (DSQ-PEM)	112/119 (94%)	13/15 (87%)	66/71 (93%)	33/33 (100%)	0.113
PEM duration (hours)					
< 1	5/114 (4%)	0/14 (0%)	4/70 (6%)	1/30 (3%)	0.476
2–3	31/114 (27%)	3/14 (21%)	23/70 (33%)	5/30 (17%)	
4–10	28/114 (25%)	2/14 (14%)	16/70 (23%)	10/30 (33%)	
11–13	8/114 (7%)	1/14 (7%)	4/70 (6%)	3/30 (10%)	
14–23	19/114 (17%)	5/14 (36%)	8/70 (11%)	6/30 (20%)	
≥ 24	23/114 (20%)	3/14 (21%)	15/70 (21%)	5/30 (17%)	
Short Form-36 Health Survey (SF-36)					
Physical functioning	60.0 (45.0–75.0)	55.0 (50.0–87.5)	60.0 (40.0–75.0)	60.0 (50.0–75.0)	0.499
Role physical	0.0 (0.0–25.0)	25.0 (0.0–62.5)	0.0 (0.0–25.0)	0.0 (0.0–25.0)	0.067
Role emotional	100.0 (33.3–100.0)	66.7 (50.0–100.0)	100.0 (0.0–100.0)	66.7 (33.3–100.0)	0.597
Vitality	25.0 (10.0–35.0)	25.0 (10.0–50.0)	25.0 (15.0–33.8)	20.0 (10.0–30.0)	0.734
Mental health	64.0 (49.0–80.0)	80.0 (62.0–80.0)	66.5 (48.0–79.0)	56.0 (48.0–72.0)	0.151
Social functioning	50.0 (25.0–75.0)	62.5 (37.5–75.0)	50.0 (25.0–75.0)	50.0 (25.0–62.5)	0.656
Bodily pain	41.0 (22.0–70.0)	41.0 (27.0–81.0)	41.0 (22.0–71.5)	32.0 (31.0–62.0)	0.882
General health	32.7 (25.0–48.8)	35.0 (25.0–53.5)	32.5 (25.5–48.8)	31.2 (30.0–45.0)	0.939
Physical Component Summary (SF36-PCS)	31.0 (27.2–36.1)	32.1 (27.8–44.9)	30.0 (28.0–33.8)	32.2 (27.1–38.8)	0.655
Mental Component Summary (SF36-MCS)	44.2 (35.1–51.0)	41.9 (39.5–47.9)	47.2 (35.1–53.1)	40.1 (34.1–51.0)	0.646
Composite Autonomic Symptom Score (COMPASS 31)					
Orthostatic intolerance	20.0 (12.0–28.0)	18.0 (9.0–24.0)	20.0 (12.0–32.0)	16.0 (12.0–20.0)	0.178
Vasomotor	0.0 (0.0–0.0)	0.0 (0.0–1.9)	0.0 (0.0–0.0)	0.0 (0.0–0.0)	0.430
Secretomotor	2.1 (0.0–4.3)	2.1 (0.0–6.4)	0.0 (0.0–4.3)	4.3 (0.0–4.3)	0.186
Gastrointestinal	5.4 (1.8–7.1)	5.4 (1.8–5.4)	4.9 (1.8–7.1)	5.4 (2.7–9.8)	0.292
Bladder	0.0 (0.0–0.0)	0.0 (0.0–0.0)	0.0 (0.0–0.0)	0.0 (0.0–1.1)	0.019
Pupillomotor	1.3 (0.3–2.3)	0.7 (0.0–1.0)	1.7 (0.5–2.3)	1.6 (1.0–2.3)	0.016
Total Score (C31 Total Score)	27.8 (17.8–38.8)	20.0 (11.0–35.9)	28.7 (20.2–40.9)	27.5 (19.2–37.9)	0.382
Munich Berlin Symptom Questionnaire (MBSQ)					
Canadian Consensus Criteria (CCC)	22/116 (19%)	3/15 (20%)	10/69 (14%)	9/32 (28%)	0.694
Institute of Medicine (IOM) criteria	29/116 (25%)	3/15 (20%)	15/69 (22%)	11/32 (34%)	> 0.999
Pediatric Case Definition (PCD-J)	10/84 (12%)	3/15 (20%)	7/69 (10%)	-	0.374
Clinical Diagnostic Worksheet (CDW-R)	10/84 (12%)	3/15 (20%)	7/69 (10%)	-	0.374
Diagnoses					
ME/CFS (ICD10-GM G93.3)	29/120 (24%)	3/16 (19%)	14/71 (20%)	12/33 (36%)	0.157
PoTS (ICD10-GM G90.8)	25/120 (21%)	3/16 (19%)	18/71 (25%)	4/33 (12%)	0.378

*ME/CFS* myalgic encephalomyelitis/chronic fatigue syndrome, *PEM* post-exertional malaise, *PoTS* postural orthostatic tachycardia syndrome

^a^Data are expressed as No. (%), median (IQR), or mean (SD), as indicated. Percentages may not total 100% because of rounding

^b^*P*-values were calculated using the *χ*^2^ test, Fisher’s exact test, or Kruskal–Wallis test, depending on the distribution and group size

Median Bell Score was 40% (IQR 30–60) (Table 3). Less than 50% or no participation in school or work was reported by 40/119 (34%) and 29/119 (24%) patients, respectively. SF-36 scores were markedly reduced compared with a German normative sample (aged 14–20 years, *N* = 228) [57], with a median PCS 31.0 (IQR 27.2–36.1) versus 57.1 (IQR 54.6–58.8) (*P* < 0.025) and a median MCS 44.2 (IQR 35.1–51.0) versus 53.5 (IQR 50.4–55.9) (*P* < 0.114) (Table 3). PoTS (ICD-10-GM G90.80) was diagnosed in 25/120 (21%) patients.

### Sub-group comparison of ME/CFS vs. non-ME/CFS patients

ME/CFS (ICD-10-GM G93.3) was confirmed or probable in 29/120 (24.2%) patients and increased with age (children 19%, adolescents 20%, adults 36%) (Table 3).

Compared with non-ME/CFS patients, those with ME/CFS had more acute COVID-19 symptoms (*P* = 0.021), more PCC symptoms (*P* = 0.032), higher FSS scores (*P* = 0.012), and more frequent PEM duration ≥ 24 h. They also showed higher COMPASS 31 total scores (*P* = 0.032), lower Bell Scores (*P* < 0.001), and lower SF36-PCS (*P* = 0.025) and SF36-MCS scores (*P* = 0.114) (Suppl. Table S2).

### Risk factor analysis using distinct clinical outcomes

Possible associations of individual candidate predictor variables during the acute phase of the SARS-CoV-2 infection and distinct outcomes at the time of PCC diagnoses were investigated: (i) *Acute OI* was significantly associated with a higher FSS score (*P* = 0.042), a higher COMPASS 31 total score (*P* < 0.001), and a lower SF36-MCS (*P* = 0.008); (ii) *the presence of OI or GI symptoms* was significantly associated with a higher number of PCC symptoms (OI: *P* < 0.001; GI: *P* < 0.001); (iii) *hospitalization due to COVID-19* was significantly associated with lower SF36-MCS (*P* = 0.048); (iv) *a higher number of COVID-19 symptoms* were significantly associated with higher FSS scores (*P* < 0.001), a higher COMPASS 31 total score (*P* = 0.010), a lower SF36-PCS (*P* = 0.001), and a higher number of chronic symptoms (*P* < 0.001); (v) *acute trouble concentrating* was significantly associated with a higher FSS score (*P* = 0.013), a higher SF36-PCS (*P* = 0.007), a higher number of chronic symptoms (*P* < 0.001), and a higher likelihood for a positive DSQ-PEM screening (*P* = 0.019); (vi) *older age* was significantly associated with a higher likelihood for positive PEM as indicated by the DSQ-PEM (*P* = 0.009), independent of sex; and (vii) *being female* was associated with a significantly higher likelihood of confirmed or suspected medical ME/CFS diagnosis and a significantly higher COMPASS 31 total score (*P* = 0.005). None of the acute features were significantly associated with brain fog (Table 4).

**Table 4:** Risk factor analyses

	Unadj. coefficient/Unadj. log odds ratio^b^	*P*-value^a^	Adj. coefficient/Adj. log odds ratio^b^	*P*-value^a^
Bell Score^c^				
Sex, female	4.238	0.202	-	-
Age, years	0.431	0.292	-	-
Number of acute symptoms	− 0.428	0.090	− 0.439	0.081
COVID-19 vaccination prior infection	− 0.537	0.882	0.266	0.942
Hospitalization due to COVID-19	− 7.512	0.252	− 8.991	0.176
Acute gastrointestinal symptoms^d^	− 1.426	0.700	− 0.201	0.958
Acute orthostatic symptoms^e^	− 3.391	0.323	− 3.524	0.304
Acute fatigue	7.511	0.177	7.446	0.180
Acute troubles concentrating	− 5.595	0.133	− 5.067	0.176
Infection susceptibility	− 3.406	0.398	− 3.549	0.378
Fatigue Severity Scale^c^				
Sex, female	− 0.141	0.416	-	-
Age, years	0.004	0.862	-	-
Number of acute symptoms	0.045	< 0.001	0.045	< 0.001
COVID-19 vaccination prior infection	0.038	0.840	0.044	0.818
Hospitalization due to COVID-19	0.461	0.231	0.461	0.166
Acute gastrointestinal symptoms^d^	0.232	0.232	0.196	0.331
Acute orthostatic symptoms^e^	0.383	0.037	0.377	0.042
Acute fatigue	− 0.040	0.895	− 0.028	0.929
Acute troubles concentrating	0.522	0.008	0.502	0.013
Infection susceptibility	− 0.214	0.314	− 0.207	0.332
COMPASS 31 Total Score^c^				
Sex, female	8.626	0.007	-	-
Age, years	0.016	0.969	-	-
Number of acute symptoms	0.620	0.014	0.633	0.010
COVID-19 vaccination prior infection	1.792	0.619	1.334	0.706
Hospitalization due to COVID-19	8.373	0.171	4.249	0.492
Acute gastrointestinal symptoms^d^	− 3.279	0.375	− 0.773	0.838
Acute orthostatic symptoms^e^	12.346	< 0.001	12.570	< 0.001
Acute fatigue	− 13.845	0.015	− 14.791	0.007
Acute troubles concentrating	4.447	0.235	5.597	0.127
Infection susceptibility	− 1.651	0.690	− 1.897	0.635
SF36–PCS^c^				
Sex, female	1.232	0.562	-	-
Age, years	− 0.005	0.984	-	-
Number of acute symptoms	− 0.514	0.001	− 0.522	0.001
COVID-19 vaccination prior infection	− 1.260	0.557	− 1.329	0.557
Hospitalization due to COVID-19	− 0.669	0.853	− 1.111	0.764
Acute gastrointestinal symptoms^d^	− 3.041	0.201	− 2.818	0.259
Acute orthostatic symptoms^e^	− 3.417	0.116	− 3.492	0.112
Acute fatigue	− 2.021	0.595	− 2.115	0.581
Acute troubles concentrating	− 6.317	0.007	− 6.323	0.007
Infection susceptibility	− 5.304	0.028	− 5.335	0.029
SF36–MCS^c^				
Sex, female	− 4.469	0.054	-	-
Age, years	− 0.978	0.730	-	-
Number of acute symptoms	− 0.337	0.059	− 0.335	0.060
COVID-19 vaccination prior infection	3.234	0.185	3.446	0.170
Hospitalization due to COVID-19	− 8.990	0.020	− 7.685	0.048
Acute gastrointestinal symptoms^d^	− 1.435	0.583	− 2.875	0.286
Acute orthostatic symptoms^e^	− 6.344	0.007	− 6.185	0.008
Acute fatigue	− 4.152	0.317	− 3.905	0.345
Acute troubles concentrating	− 4.699	0.070	− 4.788	0.063
Infection susceptibility	− 1.417	0.599	− 1.233	0.643
Number of chronic symptoms^c^				
Sex, female	4.074	0.089	-	-
Age, years	0.421	0.151	-	-
Number of acute symptoms	1.145	< 0.001	1.142	< 0.001
COVID-19 vaccination prior infection	− 1.963	0.449	− 1.303	0.618
Hospitalization due to COVID-19	6.107	0.208	4.212	0.394
Acute gastrointestinal symptoms^d^	8.422	< 0.001	9.668	< 0.001
Acute orthostatic symptoms^e^	9.314	< 0.001	9.276	< 0.001
Acute fatigue	4.536	0.341	3.802	0.425
Acute troubles concentrating	10.151	< 0.001	10.900	< 0.001
Infection susceptibility	4.284	0.144	4.121	0.158
ME/CFS diagnosis (ICD-10 GM G93.3)^c^				
Sex, female	1.234	0.014	-	-
Age, years	0.068	0.206	-	-
Number of acute symptoms	0.010	0.765	0.010	0.762
COVID-19 vaccination prior infection	− 0.692	0.206	− 0.668	0.241
Hospitalization due to COVID-19	− 1.030	0.342	− 1.433	0.191
Acute gastrointestinal symptoms^d^	0.065	0.893	0.371	0.476
Acute orthostatic symptoms^e^	− 0.423	0.341	− 0.444	0.334
Acute fatigue	− 0.923	0.144	− 0.993	0.131
Acute troubles concentrating	0.375	0.469	0.553	0.302
Infection susceptibility	− 0.256	0.647	− 0.301	0.597
PoTS diagnosis (ICD-10 GM G90.80)^c^				
Sex, female	0.372	0.426	-	-
Age, years	− 0.055	0.355	-	-
Number of acute symptoms	0.068	0.071	0.074	0.051
COVID-19 vaccination prior infection	0.231	0.639	0.140	0.782
Hospitalization due to COVID-19	1.447	0.090	1.400	0.106
Acute gastrointestinal symptoms^d^	0.047	0.926	0.195	0.718
Acute orthostatic symptoms^e^	0.249	0.606	0.353	0.476
Acute fatigue	0.178	0.829	0.111	0.894
Acute troubles concentrating	0.454	0.414	0.607	0.290
Infection susceptibility	0.755	0.144	0.748	0.152
Brain fog diagnosis (ICD-10 GM F06.7)^c^				
Sex, female	− 0.260	0.487	-	-
Age, years	0.075	0.118	-	-
Number of acute symptoms	0.005	0.850	0.002	0.942
COVID-19 vaccination prior infection	− 0.245	0.549	− 0.146	0.729
Hospitalization due to COVID-19	− 0.954	0.193	− 0.893	0.233
Acute gastrointestinal symptoms^d^	− 0.066	0.874	− 0.165	0.705
Acute orthostatic symptoms^e^	− 0.420	0.288	− 0.501	0.214
Acute fatigue	0.568	0.358	0.624	0.319
Acute troubles concentrating	0.503	0.234	0.441	0.308
Infection susceptibility	− 0.522	0.248	− 0.522	0.257
PEM^c^				
Sex, female	0.110	0.889	-	-
Age, years	0.339	0.013	-	-
Number of acute symptoms	0.160	0.021	0.152	0.033
COVID-19 vaccination prior infection	− 1.661	0.063	− 1.591	0.100
Hospitalization due to COVID-19	− 0.744	0.514	− 1.263	0.324
Acute gastrointestinal symptoms^d^	0.836	0.448	1.473	0.266
Acute OI symptoms^e^	0.939	0.235	0.819	0.330
Acute fatigue	1.366	0.129	2.125	0.047
Acute troubles concentrating	2.065	0.017	2.360	0.019
Infection susceptibility	0.654	0.554	0.593	0.605
PEM duration^c^				
Sex, female	0.005	0.988	-	-
Age, years	− 0.012	0.762	-	-
Number of acute symptoms	0.041	0.133	0.041	0.127
COVID-19 vaccination prior infection	− 0.568	0.133	− 0.623	0.104
Hospitalization due to COVID-19	− 0.106	0.872	− 0.136	0.839
Acute gastrointestinal symptoms^d^	0.270	0.467	0.317	0.406
Acute orthostatic symptoms^e^	− 0.038	0.913	− 0.033	0.927
Acute fatigue	− 0.495	0.384	− 0.506	0.376
Acute troubles concentrating	0.070	0.857	− 0.069	0.870
Infection susceptibility	− 0.069	0.870	− 0.089	0.833

*COVID-19* coronavirus disease 2019; *FSS* Fatigue Severity Scale; *GI* gastrointestinal; *ME/CFS* myalgic encephalomyelitis/chronic fatigue syndrome; *OI* orthostatic intolerance; *PCS* Physical Component Summary; *PEM* post-exertional malaise; *PoTS* postural orthostatic tachycardia syndrome; *SARS-CoV-2* severe acute respiratory syndrome coronavirus 2; *SF36-MCS* Short Form-36 Mental Component Summary; *SF36-PCS* Short Form-36 Physical Component Summary

^a^*P*-values calculated using Wald tests

^b^Adjusted models include sex and age as covariates

^c^Bell Score, FSS, COMPASS 31 Total Score, SF36-PCS, SF36-MCS, and number of chronic symptoms were modeled using multivariable linear regression. ME/CFS, PoTS, and PEM were modeled using multivariable logistic regression; PEM duration was modeled using proportional odds models

^d^Gastrointestinal symptoms included vomiting, diarrhea, or gastrointestinal complaints

^e^Orthostatic symptoms included dizziness, syncope, blackouts, or circulation dysregulation

### Risk factor analysis for severe PCC

**Fig. 1:**
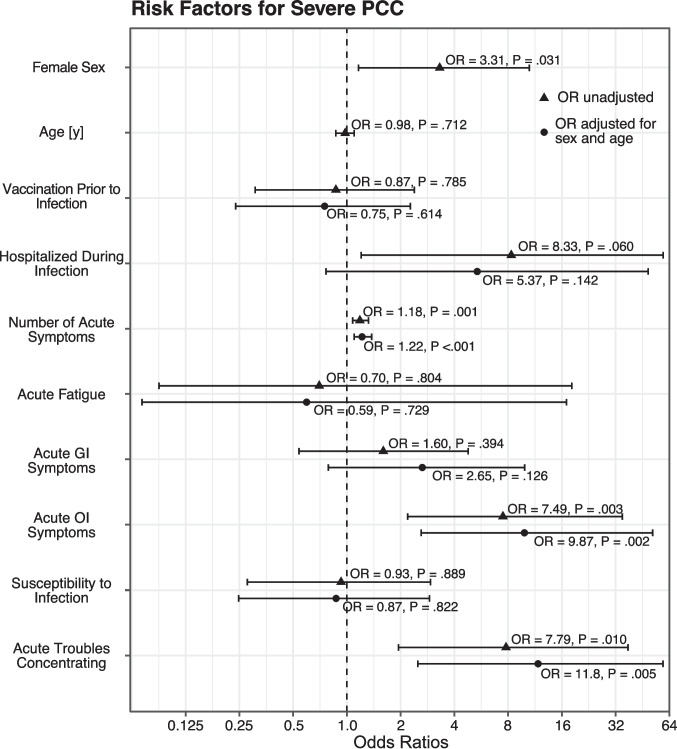
Forest plot of predictors for severe vs. moderate PCC_cyp_ (*N* = 70) derived from logistic regression models. Circles show unadjusted odds ratios (OR), triangles show OR adjusted for sex and age. Bars indicate 95% confidence intervals

The result of the* k*-means clustering is visualized in Suppl. Figure S1A. The first cluster encompassed all 29/70 (41%) patients with confirmed or probable ME/CFS and was further characterized by a lower Bell Score, more severe fatigue (FSS), more autonomic symptoms (COMPASS 31), more PCC symptoms, a lower SF36-PCS, and a lower SF36-MCS compared to the second cluster (Suppl. Figure S1B-H). This clustering was robust in sensitivity analyses excluding ME/CFS diagnosis (95.7% agreement; ARI = 0.833) and showed moderate-to-good bootstrap stability (mean Jaccard 0.82/0.83; *B* = 500). Female sex, but not age, had a significantly greater likelihood of being attributed to the severe PCC cluster (*P* = 0.031). Adjusted for sex and age, the number of acute symptoms (*P* < 0.001), acute symptoms of OI (*P* = 0.002), and acute troubles concentrating (*P* = 0.005) were significantly associated with the severe PCC cluster. (Fig. 1) Time-to-diagnosis showed no meaningful association with most retrospectively reported acute predictors, and adding time-to-diagnosis to the severity-cluster models yielded very similar effect estimates and model fits (Suppl. Tables S3 and S4).

A trained and cross-validated LASSO retained the following predictors in a final logistic regression model: female sex (coefficient: 1.08), age (coefficient: − 0.07), hospitalization during acute COVID-19 (coefficient: 0.10), number of acute COVID-19 symptoms (coefficient: 0.10), acute OI symptoms (coefficient: 1.09), acute fatigue (coefficient: − 0.95), and acute troubles concentrating (coefficient: 0.80). An ROC analysis of this model resulted in an AUC of 85.7%. The optimal threshold for predicting severe PCC was a predicted probability *P* ≥ 63.8% (Youden index; Suppl. Figure S1I). The model reliably detected CYP with “severe PCC” (sensitivity: 65.6%) or “moderate PCC” (specificity: 90.2%), yielding an overall accuracy of 80.0% (balanced accuracy: 77.9%) (Suppl. Figure S1J, K). A binary categorization of the acute number of symptoms in the penalized logistic regression model revealed that ≥ 12 acute COVID-19 symptoms had the best model fit, indicated by minimal cross-validated model error (Suppl. Figure S1L).

## Discussion

This study identified key risk factors for severe PCC in CYP after confirmed or probable COVID-19, highlighted a distinct subgroup of highly affected individuals, and introduced the MLCSQ as a standardized instrument for assessing PCC symptom burden. The key finding was that high acute symptom burden and autonomic symptoms during acute SARS-CoV-2 infection predicted severe PCC and may support timely, symptom-oriented care.

A higher number of acute symptoms were associated with greater fatigue, autonomic dysfunction, reduced HRQoL, and more chronic symptoms. This extends earlier work, showing that acute EBV-IM symptom complexity and autonomic EBV-IM symptoms predicted prolonged recovery and/or chronic fatigue [44], consistent with findings by Jason et al. linking greater symptom burden to EBV-IM-triggered ME/CFS [64].

In adults, as few as five acute COVID-19 symptoms have been associated with increased PCC risk [37]. Moreover, symptomatic versus asymptomatic SARS-CoV-2 infection has been linked to long-term sequelae in adults [65] and children [66]. Earlier pediatric studies identified hospitalization [5, 38, 39] and acute complications such as pneumonia, reduced oxygen saturation, and hypotension [58, 67] as risk factors for PCC. Our data extend this literature by suggesting that ≥ 12 acute symptoms may identify CYP at risk for severe PCC, pending external validation.

Among acute COVID-19 features, symptoms of OI emerged as a strong predictor of severe PCC and were associated with greater fatigue, autonomic distress, poorer mental health, and more PCC symptoms. This supports a central role of autonomic dysregulation in post-viral sequelae [29, 68–71].

Unsupervised clustering delineated a clinically coherent “severe” phenotype encompassing all ME/CFS cases and characterized by markedly worse fatigue, autonomic symptoms, functional impairment, and HRQoL compared with the moderate PCC cluster. The robustness of the severity cluster was supported by sensitivity clustering excluding ME/CFS diagnosis and by bootstrap stability assessment.

Female sex was associated with higher odds of severe PCC, consistent with previous pediatric reports [14, 72, 73]. In contrast, we could not confirm age as a relevant risk factor [5, 15, 16, 33, 66, 72], suggesting that more nuanced, e.g., non-linear, age-specific modeling is needed.

Acute orthostatic intolerance symptoms and acute troubles concentrating exhibited the strongest adjusted associations with severe PCC, suggesting early autonomic and neurocognitive involvement as salient signals. Acute symptom burden also tracked with risk, with the penalized model indicating ≥ 12 acute symptoms as a pragmatic cut-point warranting further validation in population-based pediatric cohorts. Acute fatigue was not discriminatory, likely due to its high prevalence across the cohort.

LASSO-penalized logistic regression selected seven independent predictors for severe PCC, including female sex, younger age, hospitalization, number of acute symptoms, acute OI symptoms, acute troubles concentrating, and absence of acute fatigue. In accordance with this, female sex has been considered a risk factor in pediatric patients [14, 72, 73]. Interestingly, acute fatigue and sex now appeared as risk factors. However, they may merely reflect an additional effect in the presence of the remaining information of the other predictors in the model. This model surely needs further evaluation in further studies.

All ME/CFS patients were assigned to the severe PCC cluster and were characterized by older age, female predominance, higher acute symptom burden, and more severe impairment across all domains. This aligns with previous studies reporting frequent autonomic dysfunction in adolescents with ME/CFS [74], and higher symptom burden in pediatric PCC patients with ME/CFS compared with non-ME/CFS patients [45].

### Strengths and limitations

Strengths of this study include the identification of a pragmatic threshold of ≥ 12 acute symptoms for early risk stratification, a large and well-characterized cohort, and a multidimensional assessment using established clinical instruments and PROMs. The data-driven analytic approach combined unsupervised clustering with LASSO and cross-validation, and the retained predictors are readily assessable in clinical practice.

This study has several limitations. First, there was no control group of CYP who fully recovered after SARS-CoV-2 infection. Risk factors identified here are therefore conditional on PCC development and not generalizable to all infected CYP. Second, the cohort represents a highly selected population referred to a single specialized fatigue center in Germany, limiting generalizability to other populations and health-care systems. Third, acute symptoms were assessed retrospectively and may be affected by recall bias, although our analyses suggest this was not a major driver of the findings. Fourth, some PROMs were originally developed for adults and are not fully validated across the pediatric age range. Fifth, missing outcome data reduced the effective sample size for clustering, regression analyses, and other analyses. Sixth, the findings of our study are not externally validated and must be considered exploratory and hypothesis-generating. Finally, prior psychiatric diagnoses, adverse life events, and socioeconomic background were not systematically assessed, so residual confounding cannot be excluded.

## Conclusions

This study highlights the importance of acute-phase symptom profiles, particularly the number of symptoms and autonomic dysfunction, for identifying CYP at risk for severe PCC. The novel MLCSQ enables a standardized, quantitative assessment of PCC symptom load. Our findings emphasize the need for systematic early symptom documentation to guide timely risk stratification, individualized follow-up, and targeted intervention in pediatric populations affected by long-term sequelae of COVID-19.

## Supplementary Information

Below is the link to the electronic supplementary material.

ESM 1 (PDF 648 KB)

## Data Availability

All data supporting the findings of this study is reported in this published manuscript and its supplementary Appendix. Ethics approval and patient consent forms do not allow individual participant data to be made publicly available. Requests for further information can be directed to the corresponding author.

## Abbreviations

AUC: Area under the receiver operating characteristic curve
ARI: Adjusted Rand index
Bell Score: Bell CFIDS disability scale
CCC: Canadian Consensus Criteria
CDW-R: Clinical diagnostic worksheet (Rowe et al.)
COMPASS-31: Composite Autonomic Symptom Score 31
COVID-19: Coronavirus disease 2019
CRF: Case Report Form
CYP: Children and young people
DGPI: German Society for Pediatric Infectious Diseases
DSQ-PEM: DePaul Symptom Questionnaire – Post-Exertional Malaise
EBV: Epstein-Barr virus
FSS: Fatigue Severity Scale
GI: Gastrointestinal
HRQoL: Health-related quality of life
ICD-10-GM: International Statistical Classification of Diseases and Related Health Problems10th revisionGerman Modification
IOM: Institute of Medicine
IQR: Interquartile range
LASSO: Least absolute shrinkage and selection operator
MCFC: Munich Chronic Fatigue Center for Young People
MBSQ: Munich Berlin Symptom Questionnaire
MCS: Mental Component Summary
ME/CFS: Myalgic encephalomyelitis/chronic fatigue syndrome
MLCSQ: Munich Long COVID Symptom Questionnaire
OI: Orthostatic intolerance
OH: Orthostatic hypotension
OR: Odds ratio
OR_adj_: Adjusted odds ratio
PASC: Post-acute sequelae of COVID-19
PCC: Post-COVID condition
PCCcyp: Post-COVID condition in children and young people
PCD-J: Pediatric case definition (Jason et al.)
PEM: Post-exertional malaise
PCR: Polymerase chain reaction
PCS: Physical Component Summary
POTS: Postural orthostatic tachycardia syndrome
PROM: Patient-reported outcome measure
ROC: Receiver operating characteristic
SARS-CoV-2: Severe acute respiratory syndrome coronavirus 2
SF-36: Short Form-36 Health Survey
TUM: Technical University of Munich
WHO: World Health Organization

## References

[1] Sørensen AIV, Spiliopoulos L, Bager P, Nielsen NM, Hansen JV, Koch A, Meder IK, Ethelberg S, Hviid A (2022) A nationwide questionnaire study of post-acute symptoms and health problems after SARS-CoV-2 infection in Denmark. Nat Commun 13(1):4213. 10.1038/s41467-022-31897-x 35864108 10.1038/s41467-022-31897-x PMC9302226

[2] Kikkenborg Berg S, Dam Nielsen S, Nygaard U, Bundgaard H, Palm P, Rotvig C, Vinggaard Christensen A (2022) Long COVID symptoms in SARS-CoV-2-positive adolescents and matched controls (LongCOVIDKidsDK): a national, cross-sectional study. Lancet Child Adolesc Health 6(4):240–248. 10.1016/S2352-4642(22)00004-9 35143771 10.1016/S2352-4642(22)00004-9 PMC8820960

[3] Borch L, Holm M, Knudsen M, Ellermann-Eriksen S, Hagstroem S (2022) Long COVID symptoms and duration in SARS-CoV-2 positive children – a nationwide cohort study. Eur J Pediatr 181(4):1597–1607. 10.1007/s00431-021-04345-z 35000003 10.1007/s00431-021-04345-z PMC8742700

[4] Stephenson T, Allin B, Nugawela MD, Rojas N, Dalrymple E, Pereira SP, Soni M, Knight M, Cheung EY, Heyman I (2022) Long COVID (post-COVID-19 condition) in children: a modified Delphi process. Arch Dis Child 107(7):674–680. 10.1136/archdischild-2021-323624 35365499 10.1136/archdischild-2021-323624 PMC8983414

[5] Morello R, Mariani F, Mastrantoni L, De Rose C, Zampino G, Munblit D, Sigfrid L, Valentini P, Buonsenso D (2023) Risk factors for post-COVID-19 condition (long COVID) in children: a prospective cohort study. EClinicalMedicine 59:101961. 10.1016/j.eclinm.2023.101961 37073325 10.1016/j.eclinm.2023.101961 PMC10101848

[6] Buonsenso D, Morello R, Mariani F, De Rose C, Mastrantoni L, Zampino G, Valentini P (2023) Risk of long COVID in children infected with Omicron or pre-Omicron SARS-CoV-2 variants. Acta Paediatr 112(6):1284–1286. 10.1111/apa.16764 36938946 10.1111/apa.16764

[7] Toepfner N, Brinkmann F, Augustin S, Stojanov S, Behrends U (2024) Long COVID in pediatrics—epidemiology, diagnosis, and management. Eur J Pediatr. 10.1007/s00431-023-05360-y 38279014 10.1007/s00431-023-05360-y PMC11001657

[8] Rao S, Gross RS, Mohandas S, Stein CR, Case A, Dreyer B, Pajor NM, Bunnell HT, Warburton D, Berg E et al (2024) Postacute sequelae of SARS-CoV-2 in children. Pediatrics. 10.1542/peds.2023-062570 38321938 10.1542/peds.2023-062570 PMC10904902

[9] World Health Organization (WHO) (2023) A clinical case definition for post COVID-19 condition in children and adolescents by expert consensus, 16 February 2023. World Health Organization, Geneva. Available at: https://iris.who.int/server/api/core/bitstreams/269e1f3a-5fa9-475b-a22d-ac4df32d9628/content

[10] Al-Aly Z, Topol E (2024) Solving the puzzle of long COVID. Science 383(6685):830–832. 10.1126/science.adl0867 38386747 10.1126/science.adl0867

[11] Lopez-Leon S, Wegman-Ostrosky T, Ayuzo Del Valle NC, Perelman C, Sepulveda R, Rebolledo PA, Cuapio A, Villapol S (2022) Long-COVID in children and adolescents: a systematic review and meta-analyses. Sci Rep 12(1):9950. 10.1038/s41598-022-13495-5 35739136 10.1038/s41598-022-13495-5 PMC9226045

[12] Sterky E, Olsson-Åkefeldt S, Hertting O, Herlenius E, Alfven T, Ryd Rinder M, Rhedin S, Hildenwall H (2021) Persistent symptoms in Swedish children after hospitalisation due to COVID-19. Acta Paediatr 110(9):2578–2580. 10.1111/apa.15999 34157167 10.1111/apa.15999 PMC8444740

[13] Molteni E, Sudre CH, Canas LS, Bhopal SS, Hughes RC, Antonelli M, Murray B, Kläser K, Kerfoot E, Chen L et al (2021) Illness duration and symptom profile in symptomatic UK school-aged children tested for SARS-CoV-2. Lancet Child Adolesc Health 5(10):708–718. 10.1016/S2352-4642(21)00198-X 34358472 10.1016/S2352-4642(21)00198-X PMC8443448

[14] Osmanov IM, Spiridonova E, Bobkova P, Gamirova A, Shikhaleva A, Andreeva M, Blyuss O, El-Taravi Y, DunnGalvin A, Comberiati P et al (2022) Risk factors for post-COVID-19 condition in previously hospitalised children using the ISARIC Global follow-up protocol: a prospective cohort study. Eur Respir J. 10.1183/13993003.01341-2021 34210789 10.1183/13993003.01341-2021 PMC8576804

[15] Zheng Y-B, Zeng N, Yuan K, Tian S-S, Yang Y-B, Gao N, Chen X, Zhang A-Y, Kondratiuk AL, Shi P-P et al (2023) Prevalence and risk factor for long COVID in children and adolescents: a meta-analysis and systematic review. J Infect Public Health 16(5):660–672. 10.1016/j.jiph.2023.03.005 36931142 10.1016/j.jiph.2023.03.005 PMC9990879

[16] Heidar Alizadeh A, Nurchis MC, Garlasco J, Mara A, Pascucci D, Damiani G, Gianino MM (2024) Pediatric post COVID-19 condition: an umbrella review of the most common symptoms and associated factors. Eur J Public Health 34(3):517–523. 10.1093/eurpub/ckae033 38411398 10.1093/eurpub/ckae033 PMC11161168

[17] Racine N, McArthur BA, Cooke JE, Eirich R, Zhu J, Madigan S (2021) Global prevalence of depressive and anxiety symptoms in children and adolescents during COVID-19: a meta-analysis. JAMA Pediatr 175(11):1142–1150. 10.1001/jamapediatrics.2021.2482 34369987 10.1001/jamapediatrics.2021.2482 PMC8353576

[18] Rao S (2024) Uncovering Long COVID in children. JAMA. 10.1001/jama.2024.13551 39167485 10.1001/jama.2024.13551

[19] Peo LC, Wiehler K, Paulick J, Gerrer K, Leone A, Viereck A, Haegele M, Stojanov S, Warlitz C, Augustin S et al (2024) Pediatric and adult patients with ME/CFS following COVID-19: a structured approach to diagnosis using the Munich Berlin Symptom Questionnaire (MBSQ). Eur J Pediatr 183(3):1265–1276. 10.1007/s00431-023-05351-z 38095713 10.1007/s00431-023-05351-z PMC10951047

[20] (2023) Correction to: Pediatric and adult patients with ME/CFS following COVID-19: A structured approach to diagnosis using the Munich Berlin Symptom Questionnaire (MBSQ). Eur J Pediatr 183:1265–1276. 10.1007/s00431-025-06112-w 10.1007/s00431-023-05351-z PMC10951047 38095713

[21] Rowe PC, Underhill RA, Friedman KJ, Gurwitt A, Medow MS, Schwartz MS, Speight N, Stewart JM, Vallings R, Rowe KS (2017) Myalgic encephalomyelitis/chronic fatigue syndrome diagnosis and management in young people: a primer. Front Pediatr 5:121. 10.3389/fped.2017.00121 28674681 10.3389/fped.2017.00121 PMC5474682

[22] Pricoco R, Meidel P, Hofberger T, Zietemann H, Mueller Y, Wiehler K, Michel K, Paulick J, Leone A, Haegele M et al (2023) One-year follow-up of young people with ME/CFS following infectious mononucleosis by Epstein-Barr virus. Front Pediatr 11:1266738. 10.3389/fped.2023.1266738 38304441 10.3389/fped.2023.1266738 PMC10830704

[23] Peter RS, Nieters A, Gopel S, Merle U, Steinacker JM, Deibert P, Friedmann-Bette B, Niess A, Muller B, Schilling C et al (2025) Persistent symptoms and clinical findings in adults with post-acute sequelae of COVID-19/post-COVID-19 syndrome in the second year after acute infection: a population-based nested case-control study. PLoS Med 22(1):e1004511. 10.1371/journal.pmed.1004511 39847575 10.1371/journal.pmed.1004511 PMC12005676

[24] Vernon SD, Zheng T, Do H, Marconi VC, Jason LA, Singer NG, Natelson BH, Sherif ZA, Bonilla HF, Taylor E et al (2025) Incidence and prevalence of post-COVID-19 myalgic encephalomyelitis: a report from the observational RECOVER-Adult study. J Gen Intern Med 40(5):1085–1094. 10.1007/s11606-024-09290-9 39804551 10.1007/s11606-024-09290-9 PMC11968624

[25] Miller CM, Borre C, Green A, Funaro M, Oliveira CR, Iwasaki A (2024) Postacute sequelae of COVID-19 in pediatric patients within the United States: a scoping review. Am J Med Open 12:100078. 10.1016/j.ajmo.2024.100078 39639960 10.1016/j.ajmo.2024.100078 PMC11617896

[26] Wulf Hanson S, Abbafati C, Aerts JG, Al-Aly Z, Ashbaugh C, Ballouz T, Blyuss O, Bobkova P, Bonsel G, Borzakova S et al (2022) Estimated global proportions of individuals with persistent fatigue, cognitive, and respiratory symptom clusters following symptomatic COVID-19 in 2020 and 2021. JAMA 328(16):1604–1615. 10.1001/jama.2022.18931 36215063 10.1001/jama.2022.18931 PMC9552043

[27] Pellegrino R, Chiappini E, Licari A, Galli L, Marseglia GL (2022) Prevalence and clinical presentation of long COVID in children: a systematic review. Eur J Pediatr 181(12):3995–4009. 10.1007/s00431-022-04600-x 36107254 10.1007/s00431-022-04600-x PMC9476461

[28] Jiang L, Li X, Nie J, Tang K, Bhutta ZA (2023) A systematic review of persistent clinical features after SARS-CoV-2 in the pediatric population. Pediatrics 152(2):e2022060351. 10.1542/peds.2022-060351 37476923 10.1542/peds.2022-060351 PMC10389775

[29] Hosozawa M, Hori M, Hayama-Terada M, Arisa I, Muto Y, Kitamura A, Takayama Y, Iso H (2024) Prevalence and risk factors of post-coronavirus disease 2019 condition among children and adolescents in Japan: a matched case-control study in the general population. Int J Infect Dis 143:107008. 10.1016/j.ijid.2024.107008 38484930 10.1016/j.ijid.2024.107008

[30] Ehm F, Tesch F, Menzer S, Loser F, Bechmann L, Vivirito A, Wende D, Batram M, Buschmann T, Ludwig M et al (2024) Long/post-COVID in children and adolescents: symptom onset and recovery after one year based on healthcare records in Germany. Infection. 10.1007/s15010-024-02394-8 39285063 10.1007/s15010-024-02394-8 PMC11825604

[31] Nittas V, Gao M, West EA, Ballouz T, Menges D, Wulf Hanson S, Puhan MA (2022) Long COVID through a public health lens: an umbrella review. Public Health Rev 43:1604501. 10.3389/phrs.2022.1604501 35359614 10.3389/phrs.2022.1604501 PMC8963488

[32] Baptista de Lima J, Salazar L, Fernandes A, Teixeira C, Marques L, Afonso C (2023) Long COVID in children and adolescents: a retrospective study in a pediatric cohort. Pediatr Infect Dis J 42(4):e109–e111. 10.1097/INF.0000000000003829 36728643 10.1097/INF.0000000000003829 PMC9990485

[33] Stephenson T, Pinto Pereira SM, Nugawela MD, McOwat K, Simmons R, Chalder T, Ford T, Heyman I, Swann OV, Fox-Smith L et al (2023) Long COVID—six months of prospective follow-up of changes in symptom profiles of non-hospitalised children and young people after SARS-CoV-2 testing: a national matched cohort study (The CLoCk study). PLoS ONE 18(3):e0277704. 10.1371/journal.pone.0277704 36877677 10.1371/journal.pone.0277704 PMC9987792

[34] Höppner J, Maier C, Schlegtendal A, Hoffmann A, Petersmann A, Lücke T, Toepfner N, Brinkmann F (2025) Long-term effects of SARS-CoV-2 infection and vaccination in a population-based pediatric cohort. Sci Rep 15(1):2921. 10.1038/s41598-024-84140-6 39849019 10.1038/s41598-024-84140-6 PMC11758015

[35] Sorg A-L, Becht S, Jank M, Armann J, von Both U, Hufnagel M, Lander F, Liese JG, Niehues T, Verjans E et al (2022) Association of SARS-CoV-2 seropositivity with myalgic encephalomyelitis and/or chronic fatigue syndrome among children and adolescents in Germany. JAMA Netw Open 5(9):e2233454. 10.1001/jamanetworkopen.2022.33454 36166227 10.1001/jamanetworkopen.2022.33454 PMC9516317

[36] Yousaf AR, Mak J, Gwynn L, Lutrick K, Bloodworth RF, Rai RP, Jeddy Z, LeClair LB, Edwards LJ, Olsho LEW et al (2025) COVID-19 vaccination and odds of post-COVID-19 condition symptoms in children aged 5 to 17 years. JAMA Netw Open 8(2):e2459672. 10.1001/jamanetworkopen.2024.59672 39992656 10.1001/jamanetworkopen.2024.59672 PMC11851240

[37] Cervia C, Zurbuchen Y, Taeschler P, Ballouz T, Menges D, Hasler S, Adamo S, Raeber ME, Bächli E, Rudiger A et al (2022) Immunoglobulin signature predicts risk of post-acute COVID-19 syndrome. Nat Commun 13(1):446. 10.1038/s41467-021-27797-1 35078982 10.1038/s41467-021-27797-1 PMC8789854

[38] Rao S, Lee GM, Razzaghi H, Lorman V, Mejias A, Pajor NM, Thacker D, Webb R, Dickinson K, Bailey LC et al (2022) Clinical features and burden of postacute sequelae of SARS-CoV-2 infection in children and adolescents. JAMA Pediatr 176(10):1000–1009. 10.1001/jamapediatrics.2022.2800 35994282 10.1001/jamapediatrics.2022.2800 PMC9396470

[39] Maddux AB, Berbert L, Young CC, Feldstein LR, Zambrano LD, Kucukak S, Newhams MM, Miller K, FitzGerald MM, He J et al (2022) Health impairments in children and adolescents after hospitalization for acute COVID-19 or MIS-C. Pediatrics. 10.1542/peds.2022-057798 35765138 10.1542/peds.2022-057798 PMC10281852

[40] Behnood SA, Shafran R, Bennett SD, Zhang AXD, O’Mahoney LL, Stephenson TJ, Ladhani SN, De Stavola BL, Viner RM, Swann OV (2022) Persistent symptoms following SARS-CoV-2 infection amongst children and young people: a meta-analysis of controlled and uncontrolled studies. J Infect 84(2):158–170. 10.1016/j.jinf.2021.11.011 34813820 10.1016/j.jinf.2021.11.011 PMC8604800

[41] Morello R, Martino L, Buonsenso D (2023) Diagnosis and management of post-COVID (Long COVID) in children: a moving target. Curr Opin Pediatr. 10.1097/MOP.0000000000001221 36660968 10.1097/MOP.0000000000001221 PMC9994801

[42] Selvakumar J, Havdal LB, Drevvatne M, Brodwall EM, Lund Berven L, Stiansen-Sonerud T, Einvik G, Leegaard TM, Tjade T, Michelsen AE et al (2023) Prevalence and characteristics associated with post-COVID-19 condition among nonhospitalized adolescents and young adults. JAMA Netw Open 6(3):e235763. 10.1001/jamanetworkopen.2023.5763 36995712 10.1001/jamanetworkopen.2023.5763 PMC10064252

[43] Lund LC, Hallas J, Nielsen H, Koch A, Mogensen SH, Brun NC, Christiansen CF, Thomsen RW, Pottegård A (2021) Post-acute effects of SARS-CoV-2 infection in individuals not requiring hospital admission: a Danish population-based cohort study. Lancet Infect Dis 21(10):1373–1382. 10.1016/S1473-3099(21)00211-5 33984263 10.1016/S1473-3099(21)00211-5 PMC8110209

[44] Bodenhausen M, Geisperger J, Lange de Luna J, Wendl J, Hapfelmeier A, Schulte-Hillen L, Pricoco R, Körber N, Bauer T, Mautner J et al (2024) Predictors of postviral symptoms following Epstein–Barr virus–associated infectious mononucleosis in young people: data from the IMMUC study. medRxiv. 10.1101/2024.05.17.24307333

[45] Jason LA, Johnson M, Torres C (2023) Pediatric post-acute sequelae of SARS-CoV-2 infection. Fatigue 11(2–4):55–65. 10.1080/21641846.2022.2162764 38044956 10.1080/21641846.2022.2162764 PMC10691585

[46] Hieber H, Pricoco R, Gerrer K, Heindrich C, Wiehler K, Mihatsch LL, Haegele M, Schindler D, Donath Q, Christa C et al (2024) The German multicenter registry for ME/CFS (MECFS-R). J Clin Med 13(11):3168 38892879 10.3390/jcm13113168 PMC11172639

[47] Jason LA, Cotler J, Islam MF, Furst J, Katz BZ (2022) Predictors for developing severe myalgic encephalomyelitis/chronic fatigue syndrome following infectious mononucleosis. J Rehabil Ther 4(1):1 35350440 10.29245/2767-5122/2021/1.1129 PMC8959090

[48] Clayton EW (2015) Beyond myalgic encephalomyelitis/chronic fatigue syndrome: an IOM report on redefining an illness. JAMA 313(11):1101–1102 25668027 10.1001/jama.2015.1346

[49] Prasser F, Kohlbacher O, Mansmann U, Bauer B, Kuhn KA (2018) Data integration for future medicine (DIFUTURE). Methods Inf Med 57(S01):e57–e65. 10.3414/ME17-02-0022 30016812 10.3414/ME17-02-0022 PMC6178202

[50] Carruthers BM, Jain AK, De Meirleir KL, Peterson DL, Klimas NG, Lerner AM, Bested AC, Flor-Henry P, Joshi P, Powles AP (2003) Myalgic encephalomyelitis/chronic fatigue syndrome: clinical working case definition, diagnostic and treatment protocols. J Chronic Fatigue Syndr 11(1):7–115

[51] Jason LA, Jordan K, Miike T, Bell DS, Lapp C, Torres-Harding S, Rowe K, Gurwitt A, De Meirleir K, Van Hoof EL (2006) A pediatric case definition for myalgic encephalomyelitis and chronic fatigue syndrome. J Chronic Fatigue Syndr 13(2–3):1–44

[52] Krupp LB, LaRocca NG, Muir-Nash J, Steinberg AD (1989) The fatigue severity scale: application to patients with multiple sclerosis and systemic lupus erythematosus. Arch Neurol 46(10):1121–1123. 10.1001/archneur.1989.00520460115022 2803071 10.1001/archneur.1989.00520460115022

[53] Cotler J, Holtzman C, Dudun C, Jason LA (2018) A brief questionnaire to assess post-exertional malaise. Diagnostics Basel. 10.3390/diagnostics8030066 30208578 10.3390/diagnostics8030066 PMC6165517

[54] Sletten DM, Suarez GA, Low PA, Mandrekar J, Singer W (2012) COMPASS 31: a refined and abbreviated composite autonomic symptom score. Mayo Clin Proc 87(12):1196–1201. 10.1016/j.mayocp.2012.10.013 23218087 10.1016/j.mayocp.2012.10.013 PMC3541923

[55] Bell D (1994) The doctor’s guide to chronic fatigue syndrome: understanding, treating, and living with CFIDS. Da Capo Press, Cambridge (MA)

[56] Ware JE Jr, Sherbourne CD (1992) The MOS 36-item short-form health survey (SF-36). I. Conceptual framework and item selection. Med Care 30(6):473–483 1593914

[57] Bellach B, Ellert U, Radoschewski F (2000) Der SF-36 im Bundes-Gesundheitssurvey: erste Ergebnisse und neue Fragen. Bundesgesundheitsblatt Gesundheitsforschung Gesundheitsschutz 43:210–216. 10.1007/s001030050036

[58] World Health Organization (WHO) (2021) Global COVID-19 clinical platform Case Report Form (CRF) for post COVID condition. World Health Organization, Geneva. Available at: https://cdn.who.int/media/docs/default-source/3rd-edl-submissions/who_crf_postcovid_feb9_2021.pdf?sfvrsn=76afd14_1&download=true

[59] Deutsche Gesellschaft für Pädiatrische Infektiologie (DGPI) (2020) DGPI Survey – Long COVID-19. Deutsche Gesellschaft für Pädiatrische Infektiologie, Berlin. Available at: https://dgpi.de/post-covid-19-survey/

[60] Funk AL, Kuppermann N, Florin TA, Tancredi DJ, Xie J, Kim K, Finkelstein Y, Neuman MI, Salvadori MI, Yock-Corrales A et al (2022) Post-COVID-19 conditions among children 90 days after SARS-CoV-2 infection. JAMA Netw Open 5(7):e2223253. 10.1001/jamanetworkopen.2022.23253 35867061 10.1001/jamanetworkopen.2022.23253 PMC9308058

[61] Morrow AK, Villatoro C, Kokorelis C, Rowe PC, Malone LA (2025) Orthostatic intolerance in children with long COVID utilizing a 10-minute passive standing test. Clin Pediatr (Phila) 64(3):416–424. 10.1177/00099228241272053 39123312 10.1177/00099228241272053 PMC12444382

[62] Brackel CLH, Lap CR, Buddingh EP, van Houten MA, van der Sande L, Langereis EJ, Bannier M, Pijnenburg MWH, Hashimoto S, Terheggen-Lagro SWJ (2021) Pediatric long-COVID: an overlooked phenomenon? Pediatr Pulmonol 56(8):2495–2502. 10.1002/ppul.25521 34102037 10.1002/ppul.25521 PMC8242715

[63] Robert Koch Institute (RKI) (2023) Weekly situation report on COVID-19, Germany, 11 May 2023. Robert Koch Institute, Berlin. Available at: https://www.rki.de/DE/Themen/Infektionskrankheiten/Infektionskrankheiten-A-Z/C/COVID-19-Pandemie/Situationsberichte/Wochenbericht/Wochenbericht_2023-05-11.pdf?__blob=publicationFile&v=1

[64] Jason LA, Cotler J, Islam MF, Sunnquist M, Katz BZ (2021) Risks for developing myalgic encephalomyelitis/chronic fatigue syndrome in college students following infectious mononucleosis: a prospective cohort study. Clin Infect Dis 73(11):e3740–e3746. 10.1093/cid/ciaa1886 33367564 10.1093/cid/ciaa1886 PMC8664491

[65] Ma Y, Deng J, Liu Q, Du M, Liu M, Liu J (2023) Long-term consequences of asymptomatic SARS-CoV-2 infection: a systematic review and meta-analysis. Int J Environ Res Public Health. 10.3390/ijerph20021613 10.3390/ijerph20021613 PMC9863678 36674367

[66] Ertesvåg NU, Iversen A, Blomberg B, Özgümüş T, Rijal P, Fjelltveit EB, Cox RJ, Langeland N (2023) Post COVID-19 condition after delta infection and omicron reinfection in children and adolescents. EBioMedicine 92:104599. 10.1016/j.ebiom.2023.104599 37149931 10.1016/j.ebiom.2023.104599 PMC10166589

[67] Floridia M, Buonsenso D, Macculi L, Weimer LE, Giuliano M, Pricci F, Bianchi L, Toraldo DM, Onder G (2024) Adolescents with persistent symptoms following acute SARS-CoV-2 infection (Long-COVID): symptom profile, clustering and follow-up symptom evaluation. Children Basel. 10.3390/children12010028 39857859 10.3390/children12010028 PMC11763728

[68] Castanares-Zapatero D, Chalon P, Kohn L, Dauvrin M, Detollenaere J, Maertens de Noordhout C, Primus-de Jong C, Cleemput I, Van den Heede K (2022) Pathophysiology and mechanism of long COVID: a comprehensive review. Ann Med 54(1):1473–1487. 10.1080/07853890.2022.2076901 35594336 10.1080/07853890.2022.2076901 PMC9132392

[69] Larsen NW, Stiles LE, Miglis MG (2021) Preparing for the long haul: autonomic complications of COVID-19. Auton Neurosci 235:102841. 10.1016/j.autneu.2021.102841 34265539 10.1016/j.autneu.2021.102841 PMC8254396

[70] Delogu AB, Aliberti C, Birritella L, De Rosa G, De Rose C, Morello R, Cambise N, Marino AG, Belmusto A, Tinti L et al (2024) Autonomic cardiac function in children and adolescents with Long COVID: a case-controlled study. Eur J Pediatr 183(5):2375–2382. 10.1007/s00431-024-05503-9 38446228 10.1007/s00431-024-05503-9 PMC11035407

[71] Kendall EK, Olaker VR, Kaelber DC, Xu R, Davis PB (2022) Association of SARS-CoV-2 infection with new-onset type 1 diabetes among pediatric patients from 2020 to 2021. JAMA Netw Open 5(9):e2233014. 10.1001/jamanetworkopen.2022.33014 36149658 10.1001/jamanetworkopen.2022.33014 PMC9508649

[72] Camporesi A, Morello R, La Rocca A, Zampino G, Vezzulli F, Munblit D, Raffaelli F, Valentini P, Buonsenso D (2024) Characteristics and predictors of Long Covid in children: a 3-year prospective cohort study. EClinicalMedicine 76:102815. 10.1016/j.eclinm.2024.102815 39296584 10.1016/j.eclinm.2024.102815 PMC11408803

[73] Miller F, Nguyen DV, Navaratnam AM, Shrotri M, Kovar J, Hayward AC, Fragaszy E, Aldridge RW, Hardelid P (2022) Prevalence and characteristics of persistent symptoms in children during the COVID-19 pandemic: evidence from a household cohort study in England and Wales. Pediatr Infect Dis J 41(12):979–984. 10.1097/INF.0000000000003715 36375098 10.1097/INF.0000000000003715 PMC9645448

[74] Stewart JM, Gewitz MH, Weldon A, Arlievsky N, Li K, Munoz J (1999) Orthostatic intolerance in adolescent chronic fatigue syndrome. Pediatrics 103(1):116–121. 10.1542/peds.103.1.116 9917448 10.1542/peds.103.1.116

