# Supplementary material for: Risk factors for severe post-COVID condition in children, adolescents, and young adults
Source: Eur J Pediatr. 2026 May 4;185(5):344. doi: 10.1007/s00431-026-06995-3 (PMC13139278; doi:10.1007/s00431-026-06995-3)
Supplement: Supplementary file 1 — (PDF 648 KB) [file 431_2026_6995_MOESM1_ESM.pdf]

SUPPLEMENTARY MATERIAL

Suppl. Table S1. *Post-COVID-Associated Diagnoses (ICD-10-GM U09.9!)*

Suppl. Table S2. *Comparison of PCC Patients with and without ME/CFS*

Suppl. Table S3. *Association between time from symptom onset to diagnosis and retrospectively reported acute-phase predictors. Each row shows the effect of time-to-diagnosis on one acute predictor in age- and sex-adjusted models. For the acute number of symptoms, estimates are linear regression coefficients (β) with 95% confidence intervals (CI). For binary acute predictors, estimates are odds ratios (OR) with 95% CI from logistic regression. P-values refer to the time-to-diagnosis term.*

Suppl. Table S4. *Sensitivity analysis for recall timing using the primary severity outcome (cluster membership). For each acute predictor, we compare age- and sex-adjusted logistic regression models without time-to-diagnosis (base model: cluster ~ age + sex + predictor) versus models additionally adjusted for time-to-diagnosis (extended model: + time-to-diagnosis). The table reports ORs (95% CI) for the acute predictor from both models, P-values, sample size, and model fit indices (AIC for base and extended models;* $\Delta AIC = AIC_{extended} - AIC_{base}$*). Δlog(OR) denotes the change in the predictor's log-odds coefficient after adding time-to-assessment.*

Suppl. Figure S1. *Clustering and prediction modelling of severe PCC outcomes*

Suppl. Material M1. *Munich Long-COVID Symptom Questionnaire (MLCSQ)*

Supplementary Table S1. *Post-COVID-associated ICD-10 GM Diagnoses (U09.9!)*

	Overall[a]<br><br>N = 120	Children[a]<br>Age 7 to 11 years<br>N = 16	Adolescents[a]<br>Age 12 to 17 years<br>N = 71	Adults[a]<br>Age 18 to 25 years<br>N = 33	P-Value[b]
R53 – Fatigue	81 / 120 (68%)	12 / 16 (75%)	51 / 71 (72%)	18 / 33 (55%)	0.170
F06 – Brain fog (F06.7)	64 / 120 (53%)	10 / 16 (63%)	31 / 71 (44%)	23 / 33 (70%)	0.034
R51 – Headache	49 / 120 (41%)	10 / 16 (63%)	26 / 71 (37%)	13 / 33 (39%)	0.160
R42 – Vertigo	32 / 120 (27%)	4 / 16 (25%)	21 / 71 (30%)	7 / 33 (21%)	0.731
R06 – Dyspnoea (R06.0)	25 / 120 (21%)	3 / 16 (19%)	16 / 71 (23%)	6 / 33 (18%)	0.948
G47 – Sleep disorders	20 / 120 (17%)	4 / 16 (25%)	10 / 71 (14%)	6 / 33 (18%)	0.502
M79 – Myalgia (M79.1)	17 / 120 (14%)	4 / 16 (25%)	7 / 71 (10%)	6 / 33 (18%)	0.199
F43 – Reaction to severe stress, and adjustment disorders	15 / 120 (13%)	1 / 16 (6%)	6 / 71 (9%)	8 / 33 (24%)	0.091
R10 – Abdominal pain (R10.4)	9 / 120 (8%)	2 / 16 (13%)	5 / 71 (7%)	2 / 33 (6%)	0.776
R11 – Nausea and vomiting	9 / 120 (8%)	4 / 16 (25%)	5 / 71 (7%)	0 / 33 (0%)	0.008
R43 – Disturbances of smell and taste (R43.8)	9 / 120 (8%)	1 / 16 (6%)	4 / 71 (6%)	4 / 33 (12%)	0.488
G47 – Hypersomnia (G47.1)	8 / 120 (7%)	1 / 16 (6%)	5 / 71 (7%)	2 / 33 (6%)	>0.999
M79 – Pain in limb (M79.6)	7 / 120 (6%)	4 / 16 (25%)	2 / 71 (3%)	1 / 33 (3%)	0.009
M25 – Pain in joint (M25.5)	6 / 120 (5%)	3 / 16 (19%)	2 / 71 (3%)	1 / 33 (3%)	0.038
R00 – Palpitations (R00.2)	5 / 120 (4%)	1 / 16 (6%)	2 / 71 (3%)	2 / 33 (6%)	0.515

a Data are presented as n/N (%).Percentages may not total 100% because of rounding.
b P-values were calculated using the Fisher exact test, or Pearson χ^2 test, as appropriate.
c Diagnoses are reported only if they occurred in ≥5 patients.
d Abbreviations: ICD-10, International Statistical Classification of Diseases and Related Health Problems, 10th Revision; PCC, pediatric post–COVID-19 condition.

Supplementary Table S2. *Comparison of PCC Patients with and without ME/CFS*

	MECFS[a] (N = 29)	non-MECFS[a] (N = 91)	P-Value[b]
Sex (female)	23/29 (79%)	48/91 (53%)	0.016
Age	15.0 (14.0 - 19.0) [0]	15.0 (13.0 - 17.0) [0]	0.224
Number of 83 possible PCC symptoms reported	33 (25.3 - 39.3) [7]	29 (19.5 - 36) [4]	0.065
Participation			0.003
full participation	0 / 28 (0%)	17 / 91 (19%)	
> 50% participation	4 / 28 (14%)	29 / 91 (32%)	
< 50% participation	12 / 28 (43%)	28 / 91 (31%)	
no participation	12 / 28 (43%)	17 / 91 (19%)	
Bell-Score	30.0 (30.0 - 50.0) [0]	50.0 (40.0 - 60.0) [2]	<0.001
Fatigue Severity Scale	6.6 (6.3 - 6.8) [3]	6.2 (5.7 - 6.6) [0]	0.012
PEM screening positive	29 / 29 (100%)	83 / 91 (91%)	0.274
PEM Duration (hours)			0.001
< 1	0 / 28 (0%)	5 / 86 (6%)	
2 - 3	2 / 28 (7%)	29 / 86 (34%)	
4 - 10	4 / 28 (14%)	24 / 86 (28%)	
11 - 13	2 / 28 (7%)	6 / 86 (7%)	
14 - 23	9 / 28 (31%)	10 / 86 (12%)	

≥ 24	11 / 28 (38%)	12 / 86 (14%)	
COMPASS 31 Total score	34.9 (25.6 - 43.5) [7]	25.6 (16.8 - 36.5) [20]	0.032
SF36-PCS	27.1 (23.6 - 32.0) [13]	31.5 (28.3 - 38.6) [21]	0.025
SF36-MCS	42.4 (29.5 - 47.9) [13]	45.6 (36.0 - 52.7) [21]	0.114
PoTS (ICD-10 GM G90.80)	9 / 29 (31%)	16 / 91 (18%)	0.278

a Data are expressed as median (IQR) or n/N (%), as appropriate. Percentages may not total 100% because of rounding. Missing data in continuous variables is presented as [n].
b P-values were calculated using the Kruskal-Wallis rank sum test, Fisher exact test, or Pearson χ^2 test, as appropriate.
c Abbreviations: ME/CFS, myalgic encephalomyelitis/chronic fatigue syndrome; PCC, pediatric post–COVID-19 condition; FSS, Fatigue Severity Scale; PEM, post-exertional malaise; SF36-PCS, SF-36 Physical Component Summary; SF36-MCS, SF-36 Mental Component Summary; PoTS, postural orthostatic tachycardia syndrome.

Supplementary Table S3. *Association between time from symptom onset to diagnosis and retrospectively reported acute-phase predictors. Each row shows the effect of time-to-diagnosis on one acute predictor in age- and sex-adjusted models. For the acute number of symptoms, estimates are linear regression coefficients (β) with 95% confidence intervals (CI). For binary acute predictors, estimates are odds ratios (OR) with 95% CI from logistic regression. P-values refer to the time-to-diagnosis term.*

**Acute predictor (recalled)**	**N**	**Effect scale**	**Effect of time (95% CI)**	**P-Value**
Number of acute symptoms	111	beta	0.007 (-0.002–0.015)	0.133
COVID-19 vaccination prior infection	111	OR	0.992 (0.987–0.996)	<0.001
Hospitalization due to COVID-19	112	OR	1.000 (0.995–1.004)	0.941
Acute gastrointestinal symptoms	111	OR	1.002 (0.999–1.005)	0.191
Acute orthostatic symptoms	111	OR	1.002 (0.999–1.005)	0.167
Acute fatigue	111	OR	1.000 (0.996–1.004)	0.901
Acute troubles concentrating	111	OR	1.002 (0.999–1.006)	0.142
Infection susceptibility	107	OR	1.002 (0.999–1.005)	0.155

Supplementary Table S4. *Sensitivity analysis for recall timing using the primary severity outcome (cluster membership). For each acute predictor, we compare age- and sex-adjusted logistic regression models without time-to-diagnosis (base model: cluster ~ age + sex + predictor) versus models additionally adjusted for time-to-diagnosis (extended model: + time-to-diagnosis). The table reports ORs (95% CI) for the acute predictor from both models, P-values, sample size, and model fit indices (AIC for base and extended models; ΔAIC = $AIC_{extended}$ − AIC_{base}). Δlog(OR) denotes the change in the predictor's log-odds coefficient after adding time-to-assessment.*

Acute predictor	N	AIC	AIC (+time)	ΔAIC	OR (base)	P-Value	OR (+time)	P-Value	Δ log(OR)
Infection susceptibility	68	94.8	94.4	−0.4	0.82 (0.245–2.773)	0.754	0.73 (0.206–2.550)	0.617	−0.127
Acute troubles concentrating	68	83.7	85.2	1.6	11.8 (2.118–64.172)	0.005	10.26 (1.808–58.210)	0.009	−0.128
Acute fatigue	68	94.8	94.5	−0.3	0.62 (0.033–11.710)	0.751	0.58 (0.031–10.946)	0.717	−0.068
Acute gastrointestinal symptoms	68	92.8	92.9	0.1	2.47 (0.711–8.588)	0.155	2.30 (0.650–8.109)	0.197	−0.074
Acute orthostatic symptoms	68	81.6	82.7	1.1	9.87 (2.435–44.577)	0.002	9.60 (2.227–41.385)	0.002	−0.028
Hospitalization due to COVID-19	68	92.2	91.8	−0.5	5.21 (0.551–49.222)	0.150	5.61 (0.581–54.183)	0.136	0.074
Number of acute symptoms	68	78.2	79.6	1.4	1.22 (1.091–1.372)	<0.001	1.22 (1.084–1.371)	<0.001	−0.004
COVID-19 vaccination prior infection	68	94.6	94.6	0.1	0.71 (0.234–2.179)	0.554	1.00 (0.296–3.347)	0.993	0.331

Suppl. Figure S1. *Clustering and prediction modelling of severe PCC outcomes*

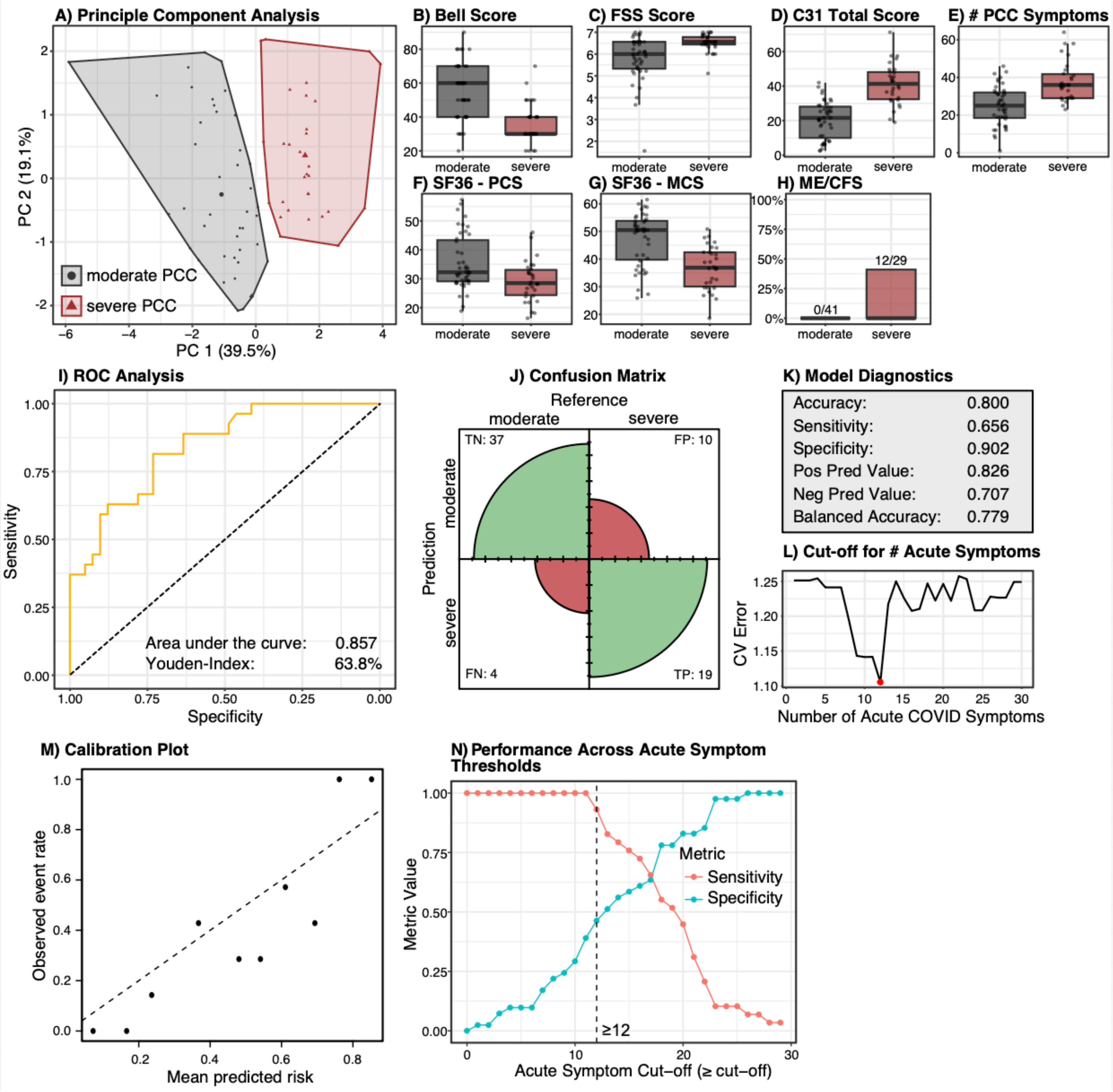

(A) Principal component analysis visualizing k-means clustering (k=2) of standardized severity-defining outcomes (Bell Score, Fatigue Severity Scale [FSS], COMPASS-31 total score [C31 Total Score], number of PCC symptoms [#PCC Symptoms], SF-36 PCS, SF-36 MCS, ME/CFS diagnosis). (B–H) Comparisons of severity-defining outcomes between clusters ("moderate PCC" vs "severe PCC"). (I) Receiver operating characteristic (ROC) curve of the LASSO-penalized logistic regression model predicting severe-cluster membership from acute-phase variables. (J) Confusion matrix at the optimal probability threshold (Youden index; p ≥ 0.638). (K) Model diagnostics of the LASSO model (cross-validation/penalty selection and fitted model). (L) Estimation of the optimal cut-off for the acute number of COVID-19 symptoms based on minimal leave-one-out cross-validation error; red dot indicates the minimum at ≥12 symptoms. (M) Calibration plot of predicted vs observed risk for severe-cluster membership. An internal bootstrap validation of the LASSO model (B=500; full modelling procedure repeated, including λ-selection) resulted in an apparent AUC of 0.857 and a Brier Score of 0.168, an optimism-corrected AUC of 0.786 and an optimism-corrected Brier Score of 0.202.

The optimism-corrected calibration intercept and slope were -0.324 and 1.168, respectively. N) Sensitivity and specificity depending on the cut-off for the acute number of symptoms, determined by the Youden Index.

Klinikum rechts der Isar   Technical University of Munich

Munich Long COVID Symptom Questionnaire (MLCSQ)

Name:<br>First Name:<br>Date of Birth:<br>Date of Completion: Completion Time: min	Name (Physician):<br>First Name (Physician):<br>Date (Physician):<br>Institution:

Please complete the questionnaire as much as possible by yourself and ask your parents for help if needed.
Any remaining open questions or issues of understanding should be clarified during the medical consultation.

		Did not occur since the SARS-CoV-2 infection	If symptoms have occurred since the SARS-CoV-2 infection, please answer the following questions: How frequently does this symptom occur?	How severe is the symptom? 1 = mild 2 = moderate 3 = severe	Medical notes
**I**	**Fatigue / Exercise intolerance**				
**1**	Fatigue (exhaustion)[1,2]	0	□ Not anymore □ Intermittent □ Persistent	1 2 3	
**2**	Worsening of symptoms following mild mental and/or physical activity (exercise intolerance)[1]	0	□ Not anymore □ Intermittent □ Persistent	1 2 3	
**3**	Reduced endurance[2]	0	□ Not anymore □ Intermittent □ Persistent	1 2 3	

**II**	**Ears-Nose-Throat (ENT)**				
**4**	Sore throat[2]	0	□ Not anymore □ Intermittent □ Persistent	1 2 3	
**5**	Swollen lymph nodes	0	□ Not anymore □ Intermittent □ Persistent	1 2 3	
**6**	Voice disorder	0	□ Not anymore □ Intermittent □ Persistent	1 2 3	
**7**	Problem swallowing[1]	0	□ Not anymore □ Intermittent □ Persistent	1 2 3	
**8**	Dryness of the mouth[3]	0	□ Not anymore □ Intermittent □ Persistent	1 2 3	
**9**	Cold[2]	0	□ Not anymore □ Intermittent □ Persistent	1 2 3	
**10**	Sinus disorder[2]	0	□ Not anymore □ Intermittent □ Persistent	1 2 3	
**11**	Loss of smell[1,2]	0	□ Not anymore □ Intermittent □ Persistent	1 2 3	
**12**	Loss of taste[1,2]	0	□ Not anymore □ Intermittent □ Persistent	1 2 3	
**13**	Altered smell[1,2]	0	□ Not anymore □ Intermittent □ Persistent	1 2 3	
**14**	Altered taste[1,2]	0	□ Not anymore □ Intermittent □ Persistent	1 2 3	
**15**	Ear pain[2]	0	□ Not anymore □ Intermittent □ Persistent	1 2 3	
**16**	Hearing impairment[1]	0	□ Not anymore □ Intermittent □ Persistent	1 2 3	
**17**	Ringing in ears[1,2]	0	□ Not anymore □ Intermittent □ Persistent	1 2 3	
**18**	Further ENT complaints:				

**III**	**Ophthalmic**				
**19**	Conjunctivitis	0	□ Not anymore □ Intermittent □ Persistent	1 2 3	
**20**	Dry eyes[3]	0	□ Not anymore □ Intermittent □ Persistent	1 2 3	
**21**	Light sensitivity[3]	0	□ Not anymore □ Intermittent □ Persistent	1 2 3	
**22**	Vision impairment[1,3]	0	□ Not anymore □ Intermittent □ Persistent	1 2 3	
**23**	Further ophthalmic complaints:				

**IV**	**Pulmonary**				
**24**	Persistent dry cough[1,2]	0	□ Not anymore □ Intermittent □ Persistent	1 2 3	
**25**	Congestion	0	□ Not anymore □ Intermittent □ Persistent	1 2 3	
**26**	Pain on breathing[1]	0	□ Not anymore □ Intermittent □ Persistent	1 2 3	
**27**	Shortness of breath at rest[1,2]	0	□ Not anymore □ Intermittent □ Persistent	1 2 3	
**28**	Shortness of breath with activity[1,2]	0	□ Not anymore □ Intermittent □ Persistent	1 2 3	
**29**	Further pulmonary complaints:				

		Did not occur since the SARS-CoV-2 infection	If symptoms have occurred since the SARS-CoV-2 infection, please answer the following questions: How frequently does this symptom occur?	How severe is the symptom? 1 = mild 2 = moderate 3 = severe	Medical notes
**V**	**Cardiovascular**				
**30**	Chest tightness[2]	0	□ Not anymore □ Intermittent □ Persistent	1 2 3	
**31**	Breath-independent chest pain[1,2]	0	□ Not anymore □ Intermittent □ Persistent	1 2 3	
**32**	Heart pounding[1,2]	0	□ Not anymore □ Intermittent □ Persistent	1 2 3	
**33**	Heart racing[1,2]	0	□ Not anymore □ Intermittent □ Persistent	1 2 3	
**34**	Orthostatic intolerance[3]	0	□ Not anymore □ Intermittent □ Persistent	1 2 3	
**35**	Fainting and/or Blackouts[1]	0	□ Not anymore □ Intermittent □ Persistent	1 2 3	
**36**	Further cardiovascular complaints:				

**VI**	**Gastro-intestinal**				
**37**	Stomach pain[1,2,3]	0	□ Not anymore □ Intermittent □ Persistent	1 2 3	
**38**	Nausea[1,2]	0	□ Not anymore □ Intermittent □ Persistent	1 2 3	
**39**	Vomiting[1,2]	0	□ Not anymore □ Intermittent □ Persistent	1 2 3	
**40**	Diarrhoea[1,2,3]	0	□ Not anymore □ Intermittent □ Persistent	1 2 3	
**41**	Constipation[1,3]	0	□ Not anymore □ Intermittent □ Persistent	1 2 3	
**42**	Bloatedness[3]	0	□ Not anymore □ Intermittent □ Persistent	1 2 3	
**43**	Flatulence[3]	0	□ Not anymore □ Intermittent □ Persistent	1 2 3	
**44**	Loss of appetite[1,2]	0	□ Not anymore □ Intermittent □ Persistent	1 2 3	
**45**	Weight loss[1,2]	0	□ Not anymore □ Intermittent □ Persistent	1 2 3	
**46**	Further gastro-intestinal complaints:				

**VII**	**Musculoskeletal**				
**47**	Muscle pain[1,2]	0	□ Not anymore □ Intermittent □ Persistent	1 2 3	
**48**	Muscle weakness[1]	0	□ Not anymore □ Intermittent □ Persistent	1 2 3	
**49**	Muscle twitching[1]	0	□ Not anymore □ Intermittent □ Persistent	1 2 3	
**50**	Muscle tremor[1]	0	□ Not anymore □ Intermittent □ Persistent	1 2 3	
**51**	Muscle stiffness[1]	0	□ Not anymore □ Intermittent □ Persistent	1 2 3	
**52**	Joint pain[1,2]	0	□ Not anymore □ Intermittent □ Persistent	1 2 3	
**53**	Joint swelling[1,2]	0	□ Not anymore □ Intermittent □ Persistent	1 2 3	
**54**	Redness and/or overheating of joints[2]	0	□ Not anymore □ Intermittent □ Persistent	1 2 3	
**55**	Back pain	0	□ Not anymore □ Intermittent □ Persistent	1 2 3	
**56**	Further musculoskeletal complaints:				

**VIII**	**Neurological**				
**57**	Headaches[1,2]	0	□ Not anymore □ Intermittent □ Persistent	1 2 3	
**58**	Trouble in concentrating[1,2]	0	□ Not anymore □ Intermittent □ Persistent	1 2 3	
**59**	Forgetfulness[1,2]	0	□ Not anymore □ Intermittent □ Persistent	1 2 3	
**60**	Light-headedness[1]	0	□ Not anymore □ Intermittent □ Persistent	1 2 3	
**61**	Dizziness[1,2]	0	□ Not anymore □ Intermittent □ Persistent	1 2 3	
**62**	Numbness or tingling[1,2]	0	□ Not anymore □ Intermittent □ Persistent	1 2 3	
**63**	Can't feel one side of body or face[1]	0	□ Not anymore □ Intermittent □ Persistent	1 2 3	
**64**	Can't move one side of body or face [1]	0	□ Not anymore □ Intermittent □ Persistent	1 2 3	
**65**	Slowness of movement[1]	0	□ Not anymore □ Intermittent □ Persistent	1 2 3	
**66**	Problems with balance[1]	0	□ Not anymore □ Intermittent □ Persistent	1 2 3	
**67**	Problems with gait/falls[1]	0	□ Not anymore □ Intermittent □ Persistent	1 2 3	
**68**	Seizures[1]	0	□ Not anymore □ Intermittent □ Persistent	1 2 3	
**69**	Further neurological complaints:				

**IX**	**Body Temperature**				
**70**	Increased sweating[2,3]	0	□ Not anymore □ Intermittent □ Persistent	1 2 3	
**71**	Fever > 38,0 °C[1,2]	0	□ Not anymore □ Intermittent □ Persistent	1 2 3	
**72**	Chills	0	□ Not anymore □ Intermittent □ Persistent	1 2 3	
**73**	Further body temperature complaints:				

		Did not occur since the SARS-CoV-2 infection	If symptoms have occurred since the SARS-CoV-2 infection, please answer the following questions: How frequently does this symptom occur?	How severe is the symptom? 1 = mild 2 = moderate 3 = severe	Medical notes
**X**	**Dermatological**				
**74**	Skin rash[1,2]	0	□ Not anymore □ Intermittent □ Persistent<br>Affected areas: □ Face □ Buttocks □ Legs □ Toes □ Trunk □ Arms □ Fingers	1 2 3	
**75**	COVID toes (purple, pink, bluish)[1,2,3]	0	□ Not anymore □ Intermittent □ Persistent	1 2 3	
**76**	Other changes in skin color on hands and/or feet (red, white)[1,3]	0	□ Not anymore □ Intermittent □ Persistent	1 2 3	
**77**	Swollen ankles[1] / Oedemas	0	□ Not anymore □ Intermittent □ Persistent	1 2 3	
**78**	Hair loss[2]	0	□ Not anymore □ Intermittent □ Persistent	1 2 3	
**79**	Further dermatological complaints:				

**XI**	**Sleep**				
**80**	Sleeping more[1,2]	0	□ Not anymore □ Intermittent □ Persistent	1 2 3	
**81**	Sleeping less[1,2]	0	□ Not anymore □ Intermittent □ Persistent	1 2 3	
**82**	Other sleep disturbances[2]	0	□ Not anymore □ Intermittent □ Persistent	1 2 3	
**83**	Further sleep complaints:				

**XII**	**Mental health**				
**84**	Behaviour change[1]	0	□ Not anymore □ Intermittent □ Persistent	1 2 3	
**85**	Tic	0	□ Not anymore □ Intermittent □ Persistent	1 2 3	
**86**	Depressed mood[1,2]	0	□ Not anymore □ Intermittent □ Persistent	1 2 3	
**87**	Loss of interest/pleasure[1]	0	□ Not anymore □ Intermittent □ Persistent	1 2 3	
**88**	Listlessness / Lack of motivation[2]	0	□ Not anymore □ Intermittent □ Persistent	1 2 3	
**89**	Anxiety[1,2]	0	□ Not anymore □ Intermittent □ Persistent	1 2 3	
**90**	Hallucinations[1]	0	□ Not anymore □ Intermittent □ Persistent	1 2 3	
**91**	Further mental health complaints:				

**XIII**	**Urogenital**				
**92**	Problems passing urine[1,3]	0	□ Not anymore □ Intermittent □ Persistent	1 2 3	
	To be answered only by patients aged ≥ 12 years				
**93**	Painful menstruation[1]	0	□ Not anymore □ Intermittent □ Persistent	1 2 3	
**94**	Changes in sexual function[1]	0	□ Not anymore □ Intermittent □ Persistent	1 2 3	
**95**	Further urogenital complaints:				

96 Further complaints: ____

____

Adapted from:
[1] WHO. Global COVID-19 Clinical Platform Case Report Form (CRF) for Post COVID condition (Post COVID-19 CRF). 2021 Feb. Retrieved on 17.11.2021 von https://cdn.who.int/media/docs/default-source/3rd-edl-submissions/who_crf_postcovid_feb9_2021.pdf?sfvrsn=76afd14_1&download=true.
[2] DGPI. DGPI Survey – Long COVID-19. Retrieved on: 26.07.2021 von https://dgpi.de/post-covid-19-survey/.
[3] Sletten DM, Suarez GA, Low PA, Mandrekar J, Singer W. COMPASS 31: a refined and abbreviated Composite Autonomic Symptom Score. Mayo Clin Proc. 2012 Dec; 87(12):1196-201. doi: 10.1016/j.mayocp.2012.10.013.
